# FFF print defect characterization through in-situ electrical resistance monitoring

**DOI:** 10.1038/s41598-024-59053-z

**Published:** 2024-05-24

**Authors:** Heime Jonkers, Alexander Dijkshoorn, Stefano Stramigioli, Gijs Krijnen

**Affiliations:** EEMCS, Robotics and Mechatronics, University of Twente, 7522NB Enschede, The Netherlands

**Keywords:** Engineering, Electrical and electronic engineering

## Abstract

Fused filament fabrication is a popular fabrication technique. Currently there is a need for in-situ monitoring modalities to gather real-time information on prints, both for quality control and closed-loop control. Despite current advancements, effective and affordable in-situ monitoring techniques for non-destructive defect detection of voids and bonding quality are still limited. This work demonstrates in-situ monitoring of fused filament fabrication through electrical resistance measurements as an alternative to thermal and optical methods. A new, easy-to-implement setup is demonstrated which measures the electrical resistance of a conductively doped filament between the nozzle and single or multi-electrodes on the bed. Defects can be located in an unprecedented way with the use of encoded axes in combination with the observed resistance variations throughout the part. A model of the anisotropic electrical conduction is used to interpret the measurements, which matches well with the data. Warping, inter-layer adhesion, under-extrusion and overhang sagging print defects can be observed in the measurements of parts with a complex geometry, which would be difficult to measure otherwise. Altogether in-situ electrical resistance monitoring offers a tool for optimising prints by online studying the influence of the print parameters for quality assessment and it opens up possibilities for closed-loop control.

## Introduction

Additive manufacturing has shown great potential as a fabrication method in a wide range of fields due to its ability to quickly and cost-effectively produce complex, customized parts^1^. One of the most widely applied printing techniques is that of Fused Filament Fabrication (FFF), which works by extruding a molten thermoplastic from a hot end onto a build platform in a line-by-line, layer-per-layer process^1^. It offers simplicity, low cost and a wide variety of available materials and is therefore used in applications as diverse as aerospace, automotive, electronics, prosthetics, fashion, construction and education^2,3^. Despite its advantages, FFF is vulnerable to errors and failures due to uncertainties in equipment and process, limiting its consistency and time- and resource-efficiency^4,5^. These failures typically result in geometric deviations, voids, warping, compromised mechanical properties, delamination, lack of material and bad surface quality^6,7^. To fully realise the potential of FFF, these challenges need to be resolved^4,6^.

Therefore methods for monitoring the print process and measuring the part properties during printing are of the utmost importance^6,8,9^. Real-time, non-destructive in-situ monitoring modalities can be used to verify part quality and to inform closed-loop feedback. A measurement science road map for polymer-based additive manufacturing from 2016 by the National Institute of Standards and Technology specifically expressed the need for monitoring modalities with the required spatial-temporal resolution, combined with in-situ control and model integration, as well as fast and accurate big data analytics^8^. Furthermore the road map indicated that these techniques should be cost-effective, cover a large spatial area and can easily be integrated into 3D-printers. Research in this area has grown significantly since^6^. Various review articles give an overview of existing in-situ monitoring methods, e.g. thermal, optical, acoustic, vibration and force/pressure monitoring, as well as the current challenges^4,6,9,10^. These reviews show that thermal and optical methods, the ones most commonly used, have already been successfully demonstrated for defect detection and closed-loop control purposes. State-of-the-art examples of this are geometry monitoring through optical measurements with a digital twin^11^, real-time defect detection through measuring of melt pool temperature deviations^12^, real-time defect detection through comparison of combined thermal and optical imaging to a baseline^13^, real-time error detection and correction with cameras in combination with large-scale machine learning^5^ and a method to link real-time defect detection with an optical camera to structural part quality^14^.

Important disadvantages of optical and thermographic imaging methods are limited in-depth information as well as limited spatial and temporal resolution^4^. While these methods can be used to estimate the adhesion quality^9^, they only give indirect information on internal voids, layer-to-layer adhesion and bond quality, that can be difficult to interpret. X-ray computed tomography can give direct information on internal defects^4,15^, however this method is not suitable for in-situ monitoring of FFF due to costs and safety concerns^10^. Despite the advancements there is still a lack of effective and cheap in-situ monitoring techniques suitable for non-destructive fault detection that can directly measure the bond quality and presence of internal voids.

In-situ monitoring of electrical properties of prints has been proposed to fill this gap. The in-situ electrical capacitance measurement of prints for example, allows measuring the presence of inter- and intra-layer defects by means of two bed electrodes, providing a layer-by-layer quality assessment^16,17^. However, this technique requires prints to be close to the electrodes, making measurements on tall geometries impossible and essentially being a volumetric method. Furthermore for FFF it is expected that the metal nozzle significantly distorts the measurements.

A solution without the aforementioned limitations is the in-situ electrical resistance monitoring of prints with conductive filament. Conductive filaments are typically used for 3D-printing of sensors, electronics and heaters^18–21^, but can also be used for in-situ monitoring. Initial work explored measuring the electrical resistance between two electrodes on the bed^22^ and between a bed and a nozzle electrode^23–25^ to study the effect of printing parameters. This preliminary research is limited to rectangular, single or few layer prints with minor data analysis. However, it does show the viability of measuring the resistance of conductively doped filaments during printing to gain information about the bond quality, part geometry and possible defects.

This work, thus for the first time presents a full, non-destructive in-situ measurement modality by means of measuring the electrical resistance. It also demonstrates its capabilities for both simple and more complex print geometries and shows a model for easy data analysis and interpretation.

**Figure 1:**
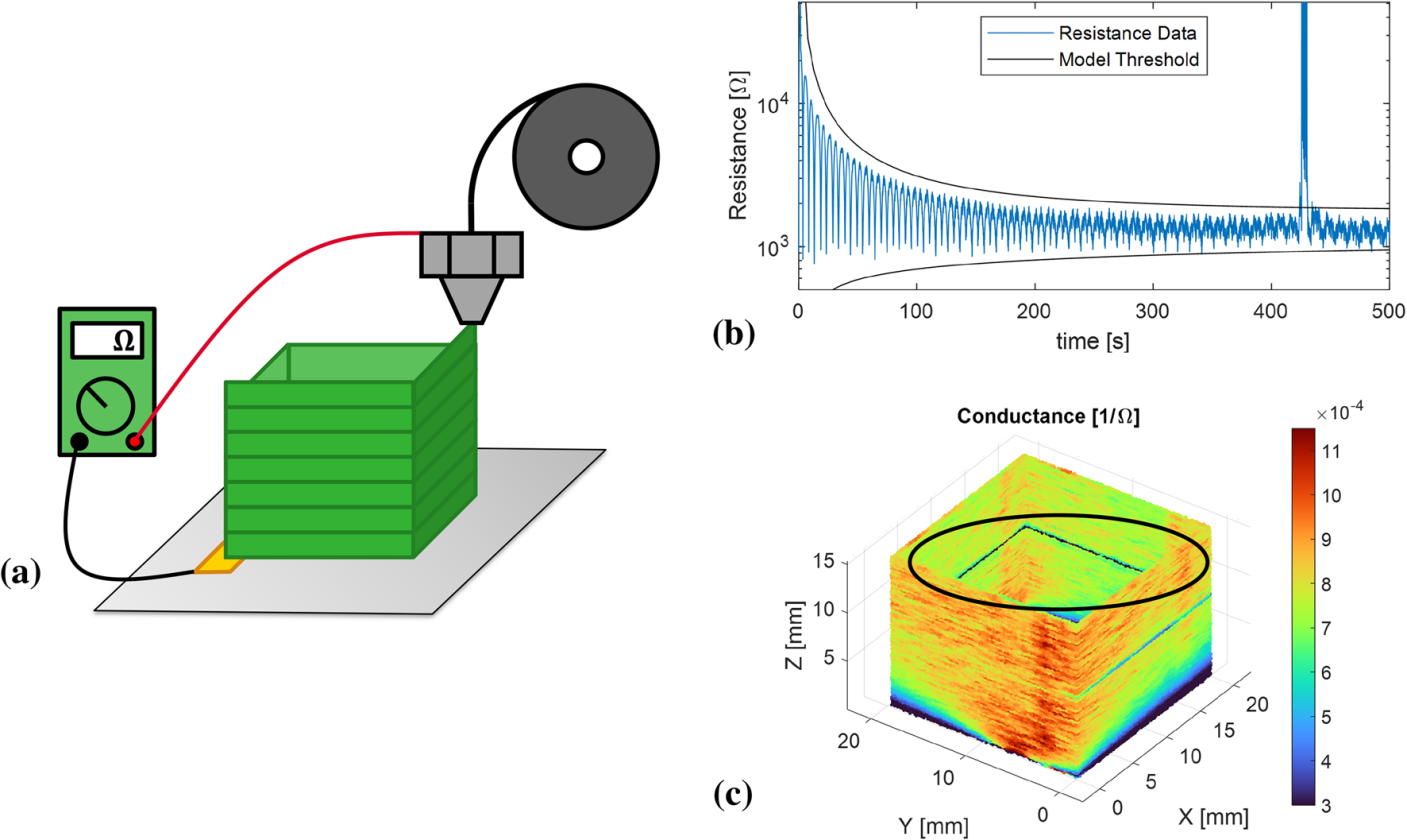
Overview of the in-situ electrical resistance monitoring methodology. (**a**) A setup with a print bed with one or more electrodes is presented for measurements, where among others square tubes are printed and monitored. (**b**) A conduction model is implemented to predict the measurement data and give a resistance threshold for defect detection. (**c**) Defects can be visualized and located through the encoded axis and resistance data.

Figure 1 presents an overview of the measurement concept. An easy-to-implement setup with a newly designed print bed is presented that can measure the electrical resistance between the nozzle and single or multiple electrodes on the print bed, Fig. 1a. Measurements are performed for a square tube and other benchmark geometries. A conduction model, based on the author’s previous work^26^, is implemented to interpret measurement data and is used to set a resistance threshold for defects, Fig. 1b. Finally, defects like poor inter-layer adhesion, overhang sagging and warping can be located with the use of encoded axes in combination with the observed resistance variations throughout the part, Fig. 1c, that would be difficult to measure otherwise.

## Results and discussion

**Figure 2:**
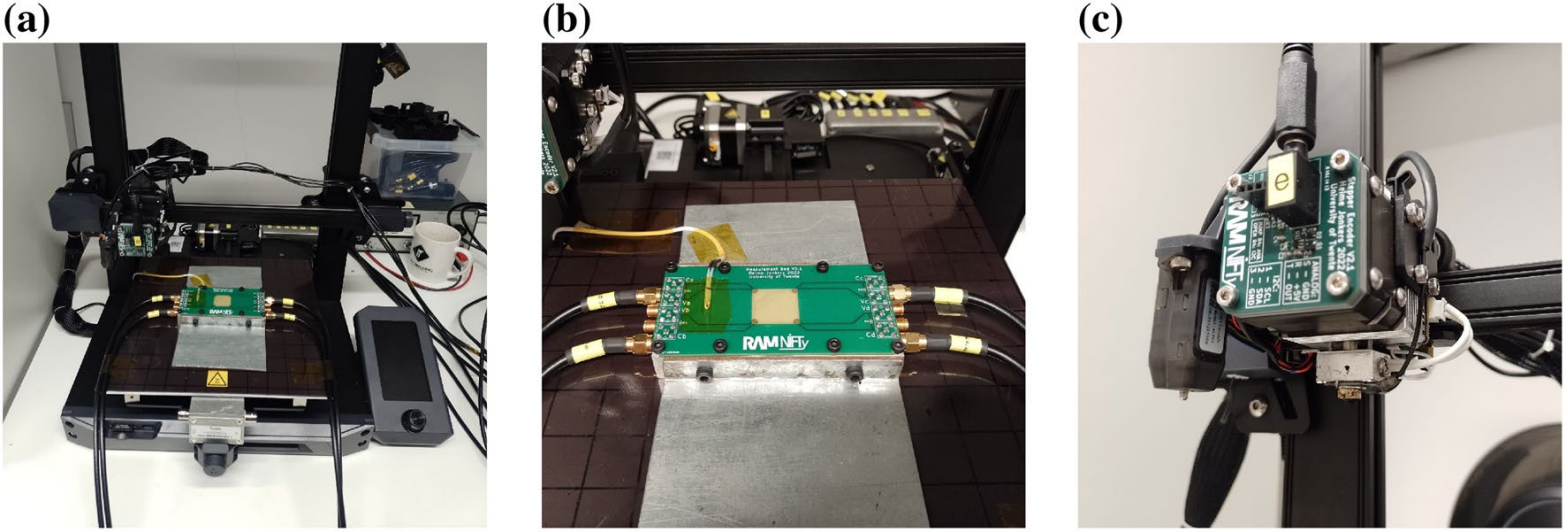
Overview of the in-situ resistance measurement platform. (**a**) Overview of the printing platform. (**b**) Custom print bed with split gold corner contacts. (**c**) Extrusion and position rotation encoder hardware.

### Setup design for the printing platform

Figure 2 provides an overview of the developed in-situ printing resistance measurement platform, based upon a commercial FFF 3D-printer (Ender S1 Pro). Resistance measurements are performed between the brass extrusion nozzle and a gold electrode equipped PCB with an exposed 20 mm $\times$ 20 mm print area. The axes and extruder of the platform have been equipped with rotary encoders on the stepper motors for monitoring the printhead position. Synchronized digital oscilloscopes are used to measure the encoder signals and the sample resistance between the bed electrodes and the nozzle. Details are provided in the methodology section at the end and in supplementary materials S.2. The method requires conductive filament, or typically filaments with a conductive filler, in this case Propopasta Conductive PLA was chosen. The in-situ measurement method has already been tested successfully with commercially available conductive PLA and conductive TPU filaments in preliminary research on simple structures with single electrode^22^. Here we demonstrate its use on more complex structures with single and multiple electrodes, relate the results to anisotropic conductivity models and show the diagnostic potential of the method.

### In-situ resistance measurements

To investigate the influence of the bed electrode configuration upon the in-situ resistance measurement, two electrode configurations were evaluated. The nozzle electrode was kept grounded while the bed electrode(s) served as current feeds in either a single corner or in parallel on all four corners. To evaluate the effects of electrode placement, a square, single-walled 20 mm $\times$ 20 mm $\times$ 20 mm tube was designed covering all corner electrodes. Three tubes were printed: one solid-walled tube, one tube with a 9 $\times$ 9 grid of 0.4 mm $\times$ 0.4 mm square holes and one tube with a 4 $\times$ 4 grid of 2.5 mm $\times$ 2.5 mm square holes. Detailed descriptions of the designed prints and slicing settings are presented in supplementary materials S.3.

**Figure 3:**
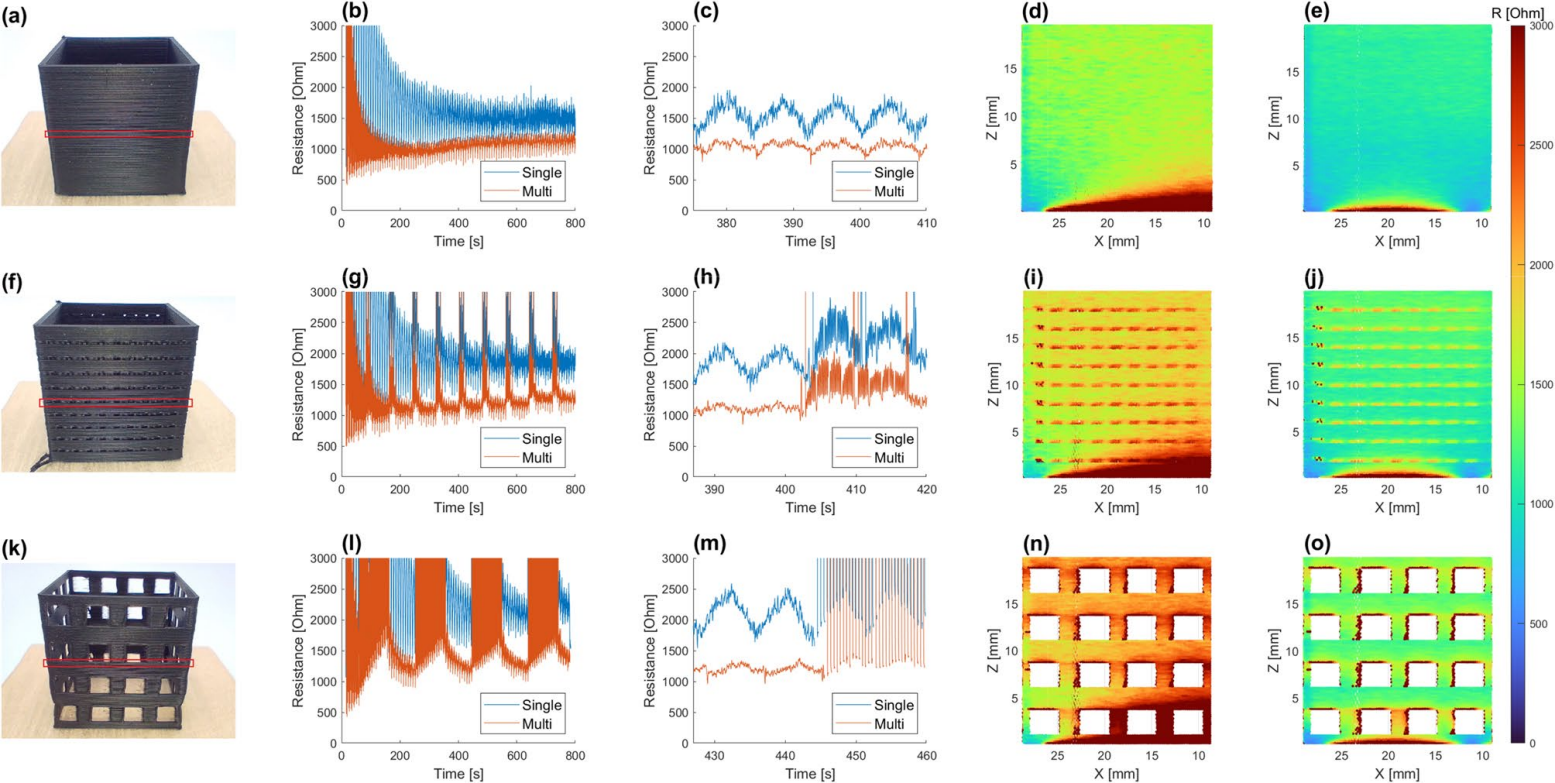
Geometric effects of electrode placement in single-walled square tube. **(a)** Solid wall design. **(f)** Small perforation design. **(k)** Large perforation design. **(b g,l)** Recorded resistance between nozzle and electrode(s) for single and multi-electrode(s). **(c,h,m)** Highlighted recorded resistance for four layers including two layers of perforations if present. **(d,i,n)** Visualization of single-electrode resistance during printing, with electrode at bottom left. **(e, j, o)** Visualization of multi-electrode resistance during printing, electrodes at bottom right and left.

In Fig. 3 an overview is presented of the square tubes, with each row representing the results for the specific design. The plotted data only displays nozzle movement during extrusion to enhance the visualization of the perforations, and is limited to a 0 $\Omega$ to 3 k$\Omega$ resistance range.

The first effect on resistance *R* is a $R\propto 1/N$ relationship due to the progression of *N* layers, where each layer adds a conduction path in parallel between the electrodes, Fig. 3b. With the progression of layers the resistance also starts to increase again, since the series resistance of the layers adds up, especially for the multi-electrode measurements. This transition point is dictated by the geometry and the electrical anisotropy of the print, as will be elaborated upon in the modeling section. These effects are also shown in the perforated tubes, Fig. 3a, b , with the gaps in between resulting in resistance spikes as an effect of the reduced contact area in these sections, as illustrated by the higher resistance for the larger gaps. The $R\propto 1/N$ relation reappears after printing on top of a layer of gaps, as an effect of the higher resistance region created by the gaps.

The difference in the geometry dependence of resistance due to single and multi-electrodes is highlighted in Fig. 3c showing four layers of the tube as indicated by the red rectangle, Fig. 3a. Each arc for the single-electrode (blue line) represents a walk of the nozzle across the perimeter of the square tube, increasing to a maximum at the outer edge from the corner electrode and then returning back to its minimum above the electrode’s corner. The multi-electrode measurement (red line) is expected to have peak resistance in between corner electrodes and minima at each corner, which can partly be observed in the resistance. In addition a slightly higher conduction is observed in the corner where the nozzle moves up to the next layer, as the effect of oozing and additional heating improves layer fusion. The suppression of the geometric effect by the multi-electrodes can clearly be seen from Fig. 3d, e, with the layer resistance becoming rather uniform after 2.5 mm for the multi-electrode measurement in comparison to the single-electrode measurement. Supplementary video S1 till S4 illustrate the single and multi-electrode tube measurements.

From Fig. 3h it becomes clear that the corner electrodes provide local minima for all resistance measurements. It also becomes apparent that the nozzle remains in contact with the sample at all perforations due to the small print gap, nevertheless resulting in a resistance spike for every perforation. The spikes can clearly be observed, locating the perforation defects in the print, Fig. 3i, j. Larger interruptions of printing, Fig. 3m, result in complete loss of contact with resistance spiking off-scale. The contact is reestablished at the pillars and the resistance has local minima above the electrodes. Breaking of the electrical contact between the nozzle and the pillars reveals itself as an increase in resistance at the edges, Fig. 3n, o. The overhanging sections of material in the first layer above the holes also have a clear resistance increase due to the printed line being the only point of electrical contact to the print.

**Figure 4:**
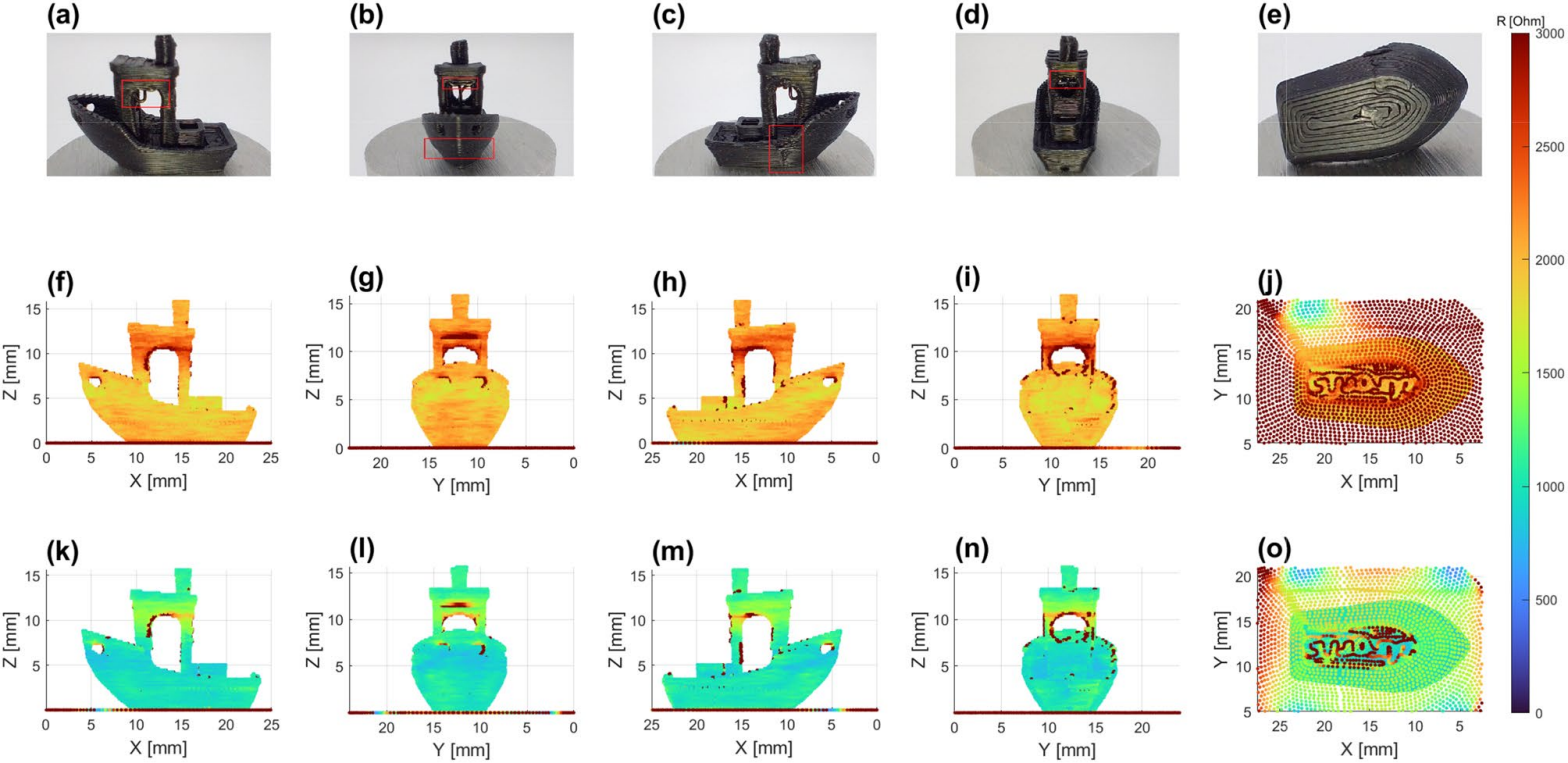
Effect of electrode placement on complex geometry realized in the Benchy standard. **(a–e)** Example print highlighting defect locations. **(f–j)** Visualization of single-electrode resistance during printing, with the electrode on the Benchy’ port back quarter. **(k–o)** Visualization of multi-electrode resistance during printing, with the electrodes on the quarters and bows.

Given the tube is a greatly simplified geometry in comparison to typical FFF printed objects, the Benchy calibration standard was scaled down to have a length of 20 mm and used as an example of single and multi-electrode measurements on a more intricate geometry, Fig. 4. In order to ensure contact to the bed electrodes a single layer brim was printed from the outside inwards, allowing for monitoring of the entire print.

Several occurring print defects can be noted from the example print, Fig. 4a–e. The first notable defects highlighted in red are the overhanging sections of the cabin arches in Fig. 4a, the rectangular windows in Fig. 4b and Fig. 4d, in both the single and multi-electrode resistance data. The second clear defect is a vertical ridge on the hull, Fig. 4c. This line is formed when the nozzle moves up to the next layer, resulting in some over-extrusion as the nozzle is parked during the *z*-movement. This ridge is not observed in the resistance data, suggesting that the inter-layer contact is near ideal for surrounding layers, given the over-extruded material does not seem to influence the conduction. If the over-extrusion would progress further, an observable conductance change would be expected, as more material would be present creating additional conduction paths.

Figure 4a–d show the deformed chimney, which is neither observed in the recorded encoder data nor in the form of discrepancies of the resistance recorded. Another feature commonly observed in the Benchy is a hull line where the infill of the deck transitions to a single wall, highlighted in red on the bottom of the curved bow, Fig. 4b. This defect can also be observed in Fig. 4f–h in the form of two lines of raised resistance. Interestingly, this effect is not seen in the multi-electrode data. Lastly, the lettering on the bottom of the Benchy as shown in Fig. 4e, j, o, was not properly formed due to the small feature size relative to the 0.4 mm nozzle. Comparison of Fig. 4j, o highlights the difference in uniformity of recorded resistances between the single and multi-electrode measurement, with the multi-electrodes capturing more detailed differences for the letter printing. Supplementary videos S5 and S6 illustrate the Benchy single and multi-electrode measurements and section S.4 shows the time and layer history of the multi-electrode Benchy measurement.

### Electrical resistance model

**Figure 5:**
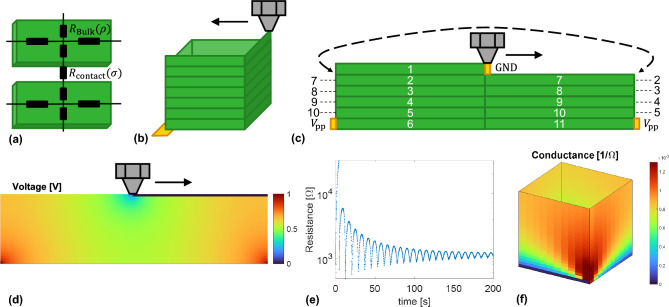
(**a**) A circuit representation of printed traxels with bulk and inter-layer resistance adapted from^26^, (**b**) printing of a single-wall square tube with a single bed electrode, (**c**) model implementation of square tube, (**d**) modeled voltage distribution, (**e**) resistance over time graph, (**f**) modeled conductance for every nozzle location in a printed square tube.

The printing process introduces electrical anisotropy^27–30^, which has to be taken into account when interpreting or predicting measurement outcomes. Therefore, the single-electrode measurements are compared with an anisotropic electrical resistance model based on earlier work^26^. At its core the model consists of an array of parallel transmission lines representing the lumped, printed track elements or traxels by coupled ordinary differential equations. They have a bulk material resistivity $\rho$ in $\Omega$m along the traxel direction and they are linked to each other via an inter-layer resistivity $\sigma$ in $\Omega \textrm{m}^{2}$, together determining the anisotropic conduction, Fig. 5a. This layer and track-based description of the model captures the main effects of the line-wise 3D-printing method and helps in understanding the effect defects have on the electrical measurements. A measure for the amount of electrical anisotropy is given by the anisotropy ratio $\Gamma$, which is 1 for the isotropic case and tends to 0 when increasing anisotropy^26^:

1 $$\begin{aligned} \Gamma := \frac{\rho }{\rho + \sigma /H} \end{aligned}$$

where *H* is the layer height that separates the interfaces.

For a square tube, Fig. 5b, the print is folded open at the electrode position to obtain a 2D representation. Every layer is split in two sections: the part where the nozzle just passed by and the part where the nozzle still needs to come along for the respective layer, Fig. 5c. The nozzle is grounded and the bed electrode is put at a positive voltage $V_{\text {pp}}$. The layers have height *H* and width *W* and the *x*-coordinate runs around the tube perimeter, where the nozzle starts above the electrode. The model calculates the voltage distribution, Fig. 5d, and the total resistance. Supplementary materials S.5 explains the detailed model implementation. The resistance at every point is determined by sweeping over the nozzle position and number of layers, Fig. 5e. The conductance is also used for plots, Fig. 5f, since defects often cause the resistance to tend towards $\infty$ (or in practice the maximum measurement value of the oscilloscope), stretching the plot and color range. Plotting the conductance values therefore spreads the data more evenly over the color scale.

The lumped resistance approximation in^26^ gives the first expectations for the model outcomes. For very few layers *N* the resistance in horizontal direction is dominant and every new layer adds a parallel current path, lowering the resistance with $R\propto 1/N$. In case of many layers or large inter-layer resistivity the resistance in the vertical direction becomes dominant, where every layer added linearly increases the resistance $R \propto N$. These scaling properties become clear when comparing single and multi-electrode measurements in Fig. 3b. The horizontal distance between electrodes for the multi-electrode lay-out is four times shorter, hence these measurements leave the $R\propto 1/N$ regime and enter the $R \propto N$ regime significantly earlier. Besides the geometry, the measured resistance is also influenced by the print settings and material properties, where a lower print temperature results in higher $\rho$ and $\sigma$^19,29,31^. Moreover the carbon-doped material has a positive temperature coefficient of resistivity (PTC) in the range of 0.01 K^-1^ 0.02^-1^^21,32,33^, hence adding heat raises the resistance^22^. Therefore in this research the samples are kept small with a big surface area to volume ratio, giving them a small thermal time constant. This makes it possible to focus on the geometry and an average $\rho$ and $\sigma$ in the model, neglecting thermal effects. Future work will focus on incorporating the thermal effects in the methodology.

**Figure 6:**
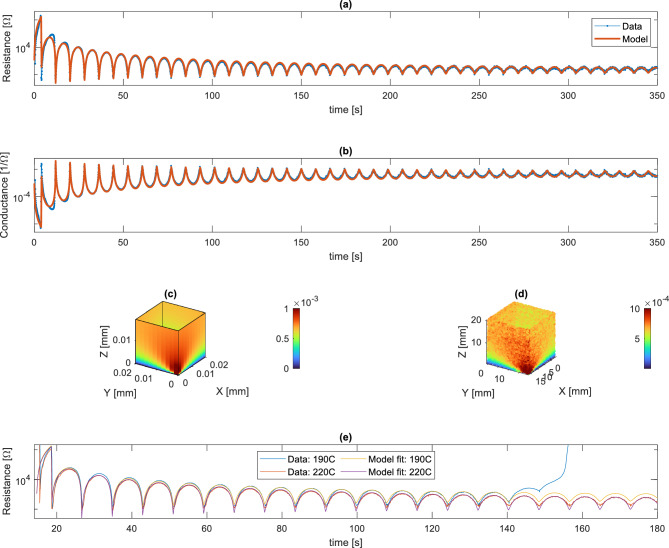
Comparison of the model and single-electrode measurements for a square tube. (**a**) Resistance over time and (**b**) conductance over time, showing good agreement except for the first few layers. Comparing the spatial (**c**) conductance model and (**d**) conductance data, showing a similar distribution. (**e**) Comparison model and measured resistance for single-electrode tube printed at nozzle temperatures of 190 ^∘^C and 220 ^∘^C. Note the resistance runaway for the 190 ^∘^C sample due to the occurrence of warping, losing contact with the bed electrode.

The model and data show good agreement, Fig. 6a–d. The model uses the print parameters from the slicer (geometry and print speed) and uses fitted electrical parameters $\rho ={0.132}\, \Omega \textrm{m}$ and $\sigma ={3 \times 10^{-6}}\, \Omega \textrm{m}^{2}$. At the start the 1/*N* regime is clearly present, leveling off towards the end. The biggest deviations between model and data occur during the first layers, which can be explained from an increase in $\rho$ and $\sigma$ due to a relatively high average sample temperature during the first layers. Besides, the effect of the nozzle position on the resistance reduces with added layers. Multi-electrode measurements for the tube or other more complex geometries can also be modeled, but for every additional bed electrode the domain needs to be split and the system of equations grows. This reduces the practical applicability of the model in the case of multi-electrodes, making Finite Element Method (FEM) simulations more effective^26,28^. A FEM analysis of the tube with large perforations is demonstrated in supplementary materials S.6. In the future perhaps FEM simulation functionality can be integrated in the slicer software, to perform an electrical conduction simulation based on the used print settings and generated print path.

To demonstrate the effect of different values for $\rho$ and $\sigma$, tubes printed with nozzle temperatures of 190 ^∘^C and 220 ^∘^C are compared in Fig. 6e. The general shapes are the same for the different temperatures, however there is a clear difference in $\rho$ and $\sigma$. For the data of the 190 ^∘^C sample the fitted parameters are $\rho ={0.14}\,\Omega \textrm{m}$ and $\sigma ={7 \times 10^{-5}}\,\Omega \textrm{m}^{2}$, resulting in significant anisotropy $\Gamma =0.29$ (compared to $\Gamma =0.90$ for the default print temperature). This indicates that the conduction model is essential in capturing the anisotropic electrical conduction for the changing geometry, where a simplified model could not achieve this. For the 220^∘^C data the fitted parameters become $\rho ={0.13}\, \Omega \textrm{m}$ and $\sigma ={8 \times 10^{-6}}\, \Omega \textrm{m}^{2}$, resulting in a lot less anisotropy $\Gamma =0.76$. Furthermore it is observed that the low temperature model warped after 140s, losing contact with the single-electrode and making the resistance run away. This shows that, in case of warping, a resistance change can be measured at the current nozzle location while it was caused somewhere else in the print.

The used model gives a lot of insight, however also has some limitations. Currently only single-walled geometries with parallel walls, homogeneous material properties and a fixed print speed can be modeled. From X-ray measurements on prints it is known that the properties are not homogeneous^15^, although single-walls are likely more homogeneous than solid prints. Moreover the model neglects thermal effects, which introduces both inhomogeneous and time-dependent material properties. Next to this the modeled contact properties are phenomenological^26^ and the value of $\sigma$ cannot be predicted yet^29^. This makes it difficult to reliably fit $\sigma$ in case of small anisotropy ($\rho H \gg \sigma$). This is not a problem however, since a small $\sigma$ also does not significantly influence the resistance. Furthermore the model and the in-situ monitoring technique are not limited to FFF, but are suitable for any other material extrusion (MEX) 3D-printing technique, like direct ink writing. In the future the model will be extended to 3D to represent more complex geometries^26^. Furthermore adaptive models for real-time digital twins will be studied, to account for the influence of defects on model parameters.

### Defect detection

The sensitivity of the method with respect to different defects is difficult to express quantitatively and heavily depends on the type of defect and the part geometry. During the first layers the resistance measurement is very sensitive to changes and defects, since added layers reduce the resistance relatively a lot, making an increase in resistance stand out. A big advantage of the method for high prints is the $R\propto N$ scaling, since the absolute resistance change stays the same for every new layer. This also means that in case of e.g. clogging, under-extrusion, sagging or warping, the resistance change is noticeable for very tall prints. Even in case of hourglass-shaped prints, like the hull-to-roof transition in the Benchy, defects can be measured without loss of detail. The constrictions only add an offset in the total resistance measurement, where on the other hand the capacitive method loses sensitivity with addition of material^16,17^. In case of prints that consist of several islands, a brim can be used to connect the parts and electrodes as was done for the Benchy.

**Figure 7:**
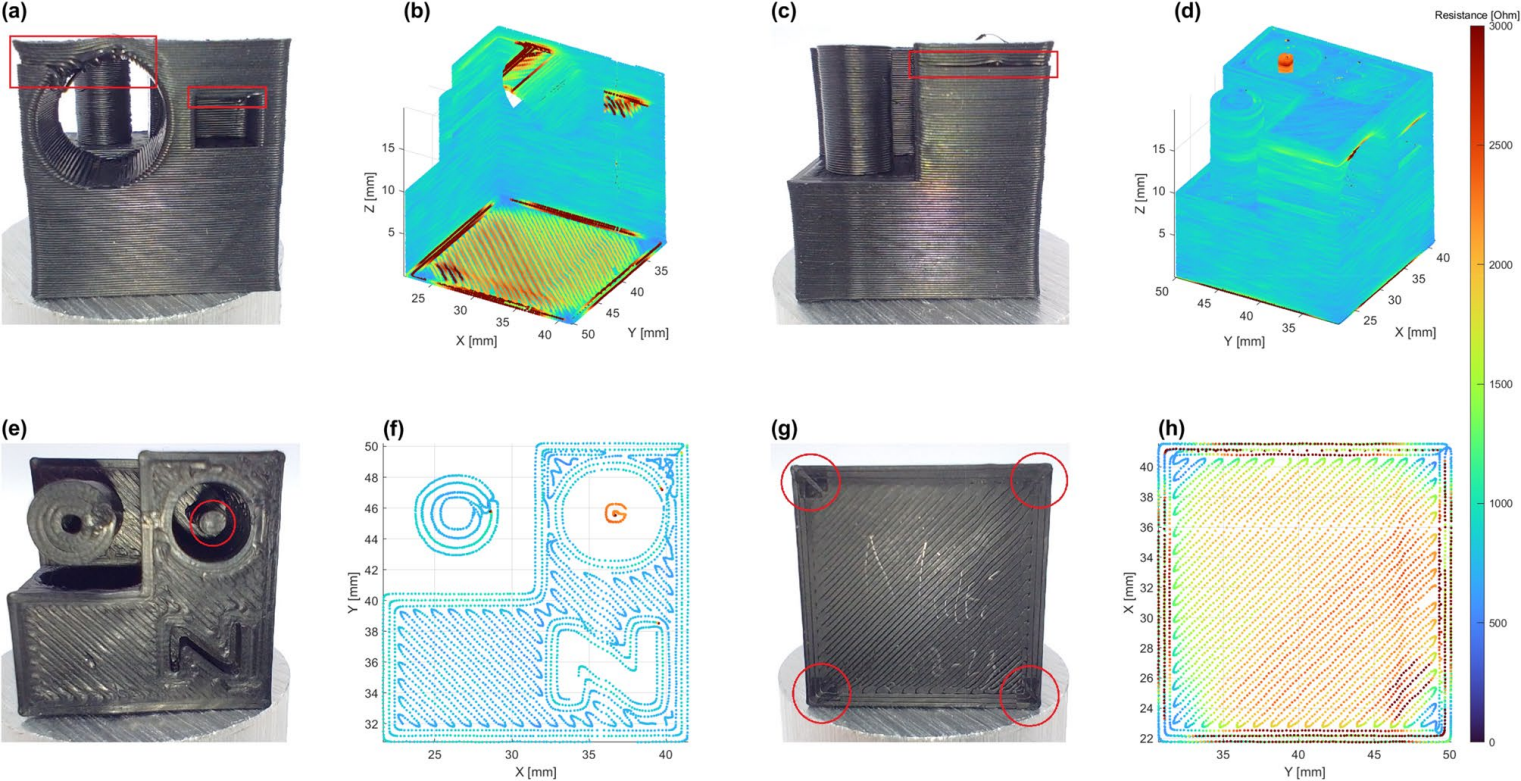
Multi-electrode resistance measurement of test object designed to highlight common print defects. **(a)** Circular and square pocket features for overhang evaluation. **(b)** Overhang defects visualized. **(c)** Side wall of circular pocket and pillar structure. **(d)** Thin pillar feature and overhang affected zone visualized. **(e)** Thin pillar, thick pillar and Z-letter feature on test object topside. **(f)** top layer resistance visualized. **(g)** First layer of test object and marks left by contact electrodes. **h** First layer resistance visualized.

In order to characterize the in-situ resistance monitoring method’s ability to detect many of the most common print defects, a test object was printed, as developed by^34^. The test object is shown in Fig. 7 and consists of several distinct features such as embedded letters on the *x*, *y*, *z*-axis, a cylindrical hole with matching cylinder and circular and square holes with an overhanging section. The data visualization and resistance range are consistent with that of the square tube and the Benchy figures.

The first detail highlighted in the test object shown in Fig. 7a, b, is the sagging overhanging section. This defect results from the lack of pushback on the nozzle by the printed filament, increasing the contact resistance between the conductive print and the nozzle. The effect of this sagging section on nearby layers is also shown in Fig. 7c, d with the edge of the hole showing an increased resistance relative to the surrounding layers. The high aspect ratio pillar is also observed to result in a high resistance, due to its small contact area relative to the surrounding print, highlighting the need for careful consideration of the print geometry for detection. The filament resistivity and the setup measurement range also influence the detection capabilities. From the estimate in supplementary materials S.7 it follows that for the measurement range of the current setup, a filament with a resistivity of 8 $\times 10^{-4} \, \Omega$m 0.73 $\Omega$m is required. This range comprises most of the commercially available, carbon-based, conductive filaments. The upper limit can easily be extended an order of magnitude with a different measurement device.

The high aspect ratio pillar along with the top layer are also highlighted in Fig. 7e, f, where the difference in contact area with respect to the larger diameter pillar stands out. Fig. 7g highlights the first layer of the print, with the red circles indicating the contact points from the split bed electrodes. In Fig. 7h the resulting resistance of the first layer is shown, reducing near the contacts as would be expected. Interestingly a step can be observed in the first layer resistance, following from the nozzle crossing the diagonal with two opposing electrodes. As the nozzle crosses the diagonal, the current suddenly has two electrodes close by, reducing the observed resistance significantly.

Several methods of defect detection would be possible on the basis of the observed resistance data. The most simple approach would be to implement threshold detection, where if the resistance crosses a certain set level, a defect is detected. This would be well suited to cover overhangs and under-extrusion defects and could possibly be extended to over-extrusion defects if a lower bound is employed. To also be able to deal with geometric effects, either a recording of a known successful print or a simulated print could be used as a baseline to set detection thresholds with a margin of error, like in^12,13^. For simple geometries the model can serve as baseline or threshold like in Fig. 1, convincingly detecting warping as shown in Fig. 6. The printing of the first layer could also be adapted in a closed-loop form, to realize a compensation of nozzle height on the basis of traxel resistance, where modeling could compensate for anisotropy and print path effects.

In future studies multiplexing can enable tomography, improving the ability to distinguish types of defects and to determine defect locations. Combined with AC measurements, this will allow for full electrical impedance tomography, where the electrical model is already implemented for AC^26^. By making use of sensor fusion with multiple monitoring modalities, the full potential of defect detection and closed-loop control can be unleashed^6^. Combined with machine learning models this data can for example be used for strength prediction in the final product^14^.

## Conclusion

In summary, the presented in-situ electrical resistance monitoring allows for assessing the printing process and detection of among others warping, poor inter-layer adhesion, under-extrusion and overhang sagging in parts with a complex geometry in an unprecedented way. Multi-electrode measurements offer the benefit of suppressing the effect of nozzle position on the resistance, improving print characterization. For selected, relatively simple structures, the conduction model matches well with the data and serves as baseline for defect detection. Altogether in-situ electrical resistance monitoring offers a tool for quality assessment and for studying the influence of print parameter settings and it opens the way for closed-loop control.

## Methods

### Printing platform

The printing platform was based upon a Ender S1 Pro modified with Marlin firmware by Miguel Risco-Castillo^35^. It was fitted with a custom 20 mm $\times$ 20 mm PCB print bed with split gold electrodes and AS5600 based magnetic rotation encoders for printhead position/extrusion. The nozzle and print bed PID control were tuned to match the new setup and data was captured with TiePie HS5 digital oscilloscopes for synchronous resistance and voltage sampling at 5 kHz in 20 M$\Omega$ and 20 V DC range respectively. Detailed descriptions of each part of the printing platform can be found in supplementary materials (S.2).

### Print slicing

Print geometries were sliced with Cura 5.2.1, with settings optimised for the use of Propopasta Conductive PLA: $T_\text {nozzle}$ was 205 ^∘^C, $T_\text {bed}$ was 60 ^∘^C, the flow multiplier was 100%. To ensure real world comparable printing a layer height of 0.2 mm, a 0.4 mm nozzle and a 8 mm brim or a three line skirt were used during printing to ensure consistent extrusion. To minimize variation in material cooling after extrusion, no fan cooling was employed. The parameters used for individual prints are reported in supplementary materials (S.3).

### Encoder post-processing

Given analog encoder outputs consisting of a 0 V to 4.5 V signal representing a single revolution of the stepper motor’s axis, unwrapping was required. This was achieved by detecting jumps, sharpening it to a 1 sample jump and then normalizing the data to 0 to $2\pi$. MATLAB(R2020b) unwrap() and medfilt() could then be used to unwrap the signal and filter it by $f_{\text {s}}/10$ samples or around 10Hz. The final step is to correct the offset on each axis by subtracting the minimal value from all data points to ensure the start point of the print is at the $x=0$ and $y=0$ position.

### Resistance post-processing

The resistance data was filtered with MATLAB(R2020b) movmean() function by $f_{\text {s}}/10$, after which the date was reduced to 1/100 samples to achieve a time step of 20 ms, translating to a movement of 0.4 mm per step at the selected 20 mm s^-1^ printing speed. The color scale selected for the visualization of the data was ’Turbo’ with the color bar being scaled to a range of 0 k$\Omega$ to 3 k$\Omega$ to highlight the most relevant resistance changes. In addition to this filtering, an extrusion rate based retraction filter was created, allowing for masking of points where no extrusion occurred.

### Model implementation

The model is implemented in Matlab (Matlab R2020a) and it is based upon the open-access code in^36^. The model is verified and validated with simple analytical models, FEM simulations and experiments in a previous study^26,28^. The geometrical parameters are taken from the slicer settings. The values for $\rho$ and $\sigma$ are fitted by hand, based on the slope and offset of the resistance data as a function of time.

### Supplementary Information

Supplementary Video 1.

Supplementary Video 2.

Supplementary Video 3.

Supplementary Video 4.

Supplementary Video 5.

Supplementary Video 6.

Supplementary Information.

## Data Availability

The datasets generated and/or analysed during the current study are available in the 4TU.ResearchData repository, https://doi.org/10.4121/c17ac579-bab1-4f58-ba67-a95182fd021f.v1.
