# Supplementary material for: FFF print defect characterization through in-situ electrical resistance monitoring
Source: Sci Rep. 2024 May 24;14:11906. doi: 10.1038/s41598-024-59053-z (PMC11126602; doi:10.1038/s41598-024-59053-z)
Supplement: Supplementary file 7 — Supplementary Information. [file 41598_2024_59053_MOESM7_ESM.pdf]

Supplementary Information: FFF print defect characterization through in-situ electrical resistance monitoring

Heime Jonkers[1,*,+], Alexander Dijkshoorn[1,+], Stefano Stramigioli[1], and Gijs Krijnen[1]

[1]University of Twente, EEMCS, Robotics and Mechatronics, Enschede, 7522NB, The Netherlands

[*]correspondence: h.r.jonkers@utwente.nl

[+]these authors contributed equally to this work

1. Supplementary Videos

- Supplementary Video S1: Data animation and camera footage of the single-electrode measurement for the square tube.
- Supplementary Video S2: Animation comparing the data of the single and multi-electrode measurements for the square tube.
- Supplementary Video S3: Animation comparing the data of the single and multi-electrode measurements for the square tube with small perforations.
- Supplementary Video S4: Animation comparing the data of the single and multi-electrode measurements for the square tube with large perforations.
- Supplementary Video S5: Data animation and camera footage of the multi-electrode measurement for the Benchy.
- Supplementary Video S6: Animation comparing the data of the single and multi-electrode measurements for the Benchy.

2. In-situ resistance measurement platform

To have a platform for in-situ resistance and position measurements during printing required the modification of a commercial Ender 3 S1 Pro FFF printer. This printer was chosen for several reasons: Firstly, due to the accessibility of its stepper motors for fitting of position encoders. Secondly, for its extrusion head construction, allowing for access to the printing nozzle, for the placement of an electrical contact on the nozzle itself. Thirdly, for its direct drive extruder allowing tight control of extrusion and compatibility with flexible and other difficult to print materials. And lastly, the relative ease at which it could be converted to make use of the open source Marlin firmware,[1] required to operate the new electrode equipped print bed and modified print head.

An overview of the modified printer is provided in Fig. 1, where Fig. 1a shows the frontside of the printer with custom extruder encoder and print bed. Fig. 1b Shows the encoder signal and power distribution box and further x, y, z-axis encoders. Fig. 1d shows the custom print bed which is equipped

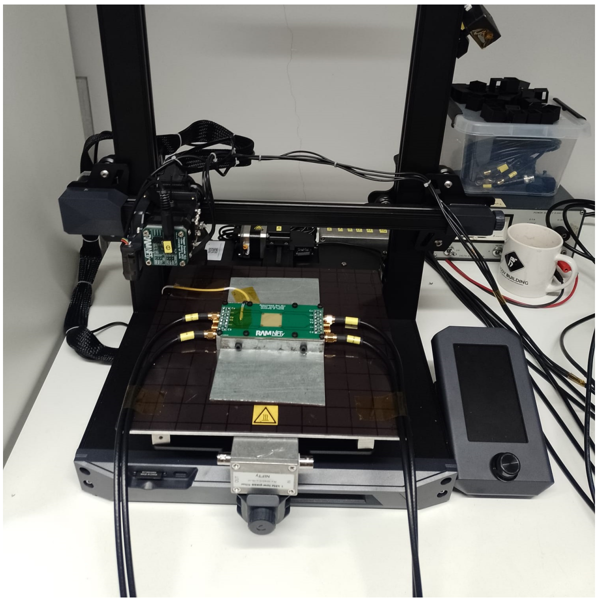

(**a**) Overview of printer

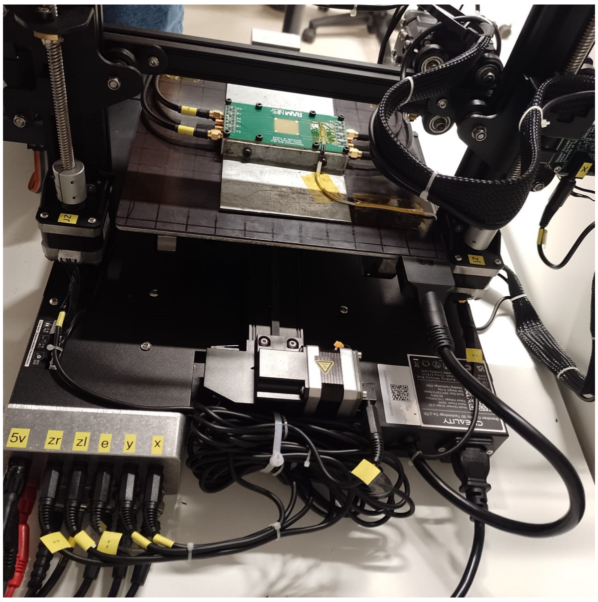

(**b**) Overview of wiring

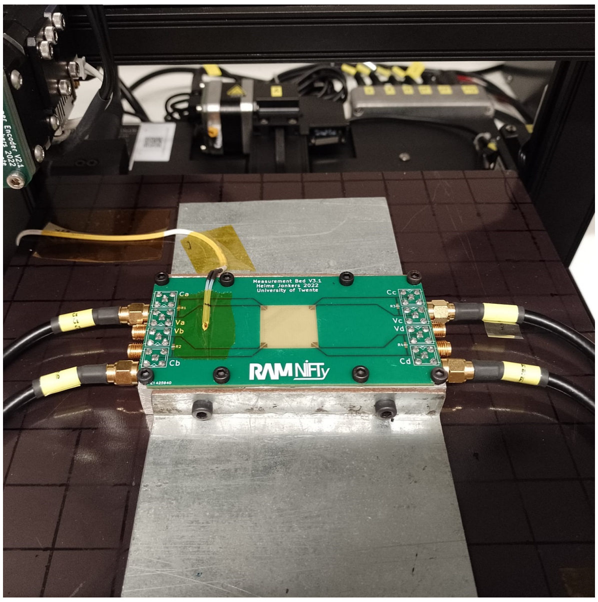

(**c**) Gold electrode equipped print bed

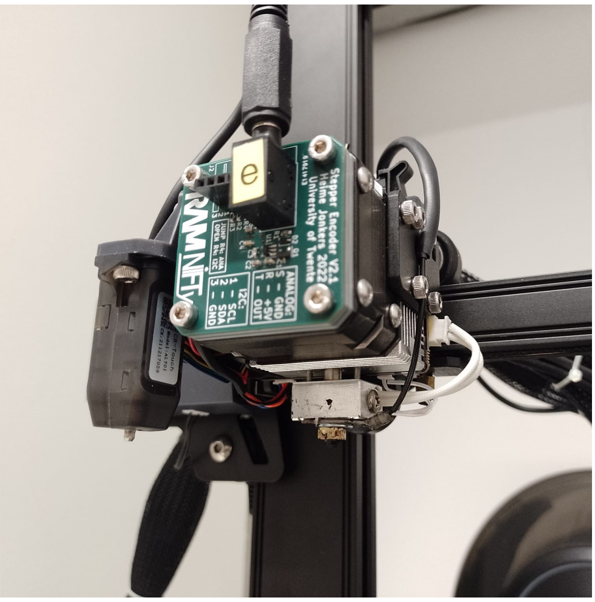

(**d**) Hall effect rotation encoder shield

Figure 1. In-situ resistance measurement platform.

with gold corner electrodes and an exposed FR-4 print area. Fig. 1d shows a close up of the print head with electrical contact and extruder encoder. The bed leveling probe on the print head is also shown, allowing for repeatable print layer heights.

2.1. Data acquisition

Measurement of the resistance between the nozzle and the bed electrodes was performed through the use of three TiePie Engineering HS5 synchronizable oscilloscopes. Data was collected with $0\,\mathrm{V}$ to $20\,\mathrm{V}$ range for the encoder axes and $0\,\mathrm{M\Omega}$ to $2\,\mathrm{M\Omega}$ range for the impedance measurement, sampled at $5\,\mathrm{kHz}$. The analog output voltage of the the encoders was connected to the TiePie oscilloscopes through standard $50\,\Omega$ coaxial cables with BNC connections, making use of a shielded breakout box to separate the signal and power supply connections. The print bed electrodes were connected to the oscilloscopes through a $50\,\Omega$ BNC to SMA cable. The nozzle was grounded through an non-shielded cable which was connected at the nozzle end with a ring terminal and connected at the oscilloscope by a BNC breakout

terminal. An overview of the electrical connections is shown in Fig. 2.

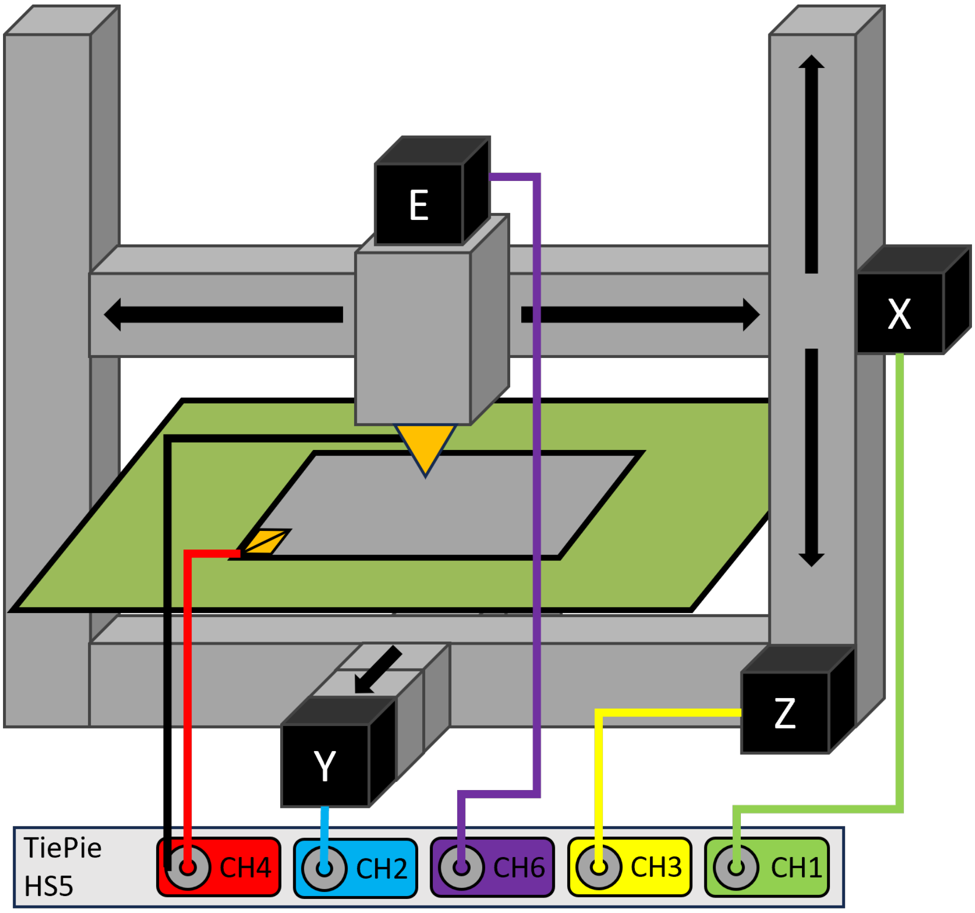

Figure 2. Wiring overview between sensorized 3D printer and TiePie oscilloscopes

2.2. *Print bed design*

As mentioned previously, the print bed of the printer was modified to contain a custom gold electrode equipped PCB. The center contains a $20\,\mathrm{mm} \times 20\,\mathrm{mm}$ area of exposed FR-4 circuit board material, with split $2\,\mathrm{mm} \times 2\,\mathrm{mm}$ gold contacts in each corner, allowing for 2- and 4-point measurements between the electrodes. In Fig. 3 a render of the designed printed circuit board is provided, where it can be observed that the current C and voltage V connections are split for each corner electrode. The exposed area in the center is also highlighted in grey, with the connecting copper traces covered in solder mask being shown in light green. The dark green area represents the FR-4 material, with the gold color representing the through-hole via's and the gold coated contacts.

The option for jumper resistors is provided to connect the split electrodes and the board was designed with SMA connectors for connection to the electrodes. The PCB was designed to not contain a copper ground plane, with the coaxial cable grounds being terminated at the digital oscilloscope. A bill of materials for the jumper resistors and the SMA connectors on the measurement PCB is provided in table1

Table 1. Bill of material for print bed

Id	Designator	Package	Quantity	Designation
1	J1,J2,J7,J5,J4,J3,J8,J6	SMA_901-143_Horizontal	8	SMA 90°
2	R2,R1,R3,R4	R_0603_1608Metric	4	0R Jumper

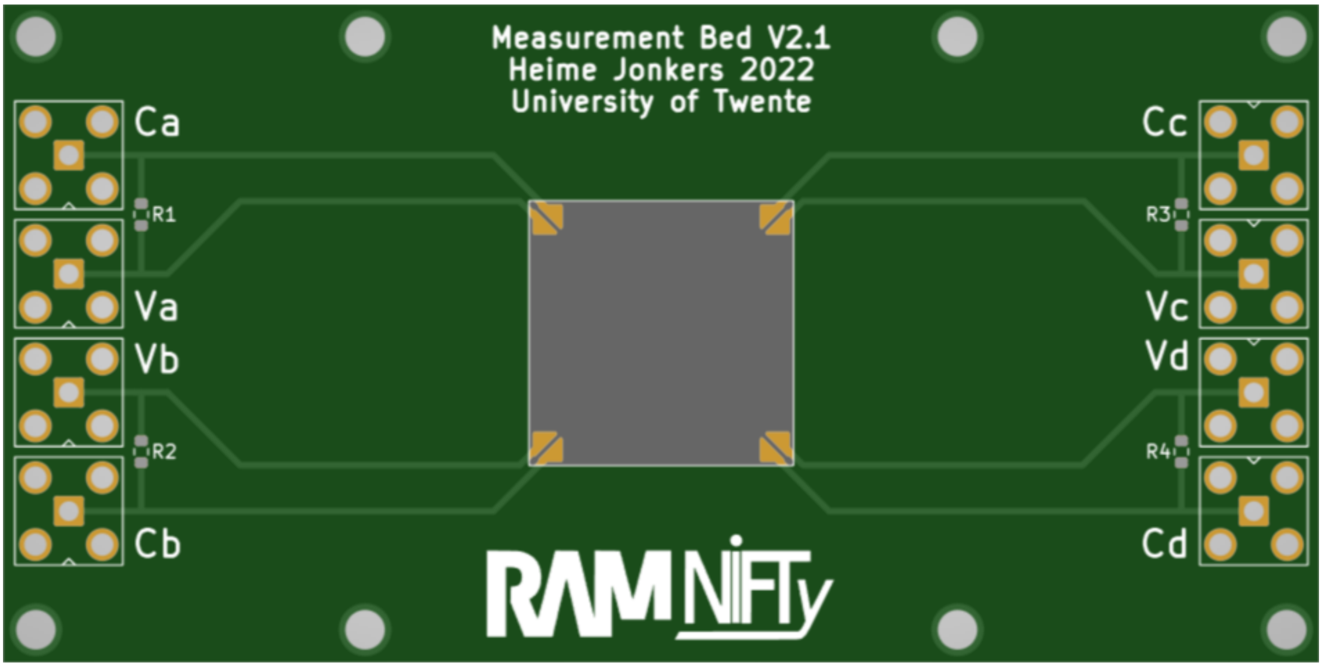

Figure 3. Print Bed circuit board layout, with solder mask free center area

The new bed was designed such that it could be stuck to the magnetic original bed of the printer, as shown in Fig. 1c.To ensure a good thermal contact to the heater of the original bed and to provide a significant thermal mass for stable bed temperatures, an aluminium plate of 20 mm thickness was used underneath the custom PCB, with the thermocouple being placed on the print surface. This change in thermal mass and response also required the re-calibration of the bed dimensions, leveling and the PID settings for the bed and nozzle heater, for which the settings can be found in Table 2. It should also be noted that the print cooling fan was removed and that the nozzle was calibrated after homing above the print bed set at a stable temperature of 60 °C. Commonly the print bed heater already consists of a custom PCB with copper traces as a heating element, allowing for simple integration of sensing electrodes in current commercially available FFF printers.

Table 2. Custom Marlin configuration settings

Bed PID		Nozzle PID		Bed size		Mesh inset		Probe offset	
Kp	137.74	Kp	16	x	40 mm	x	[10,30]	x	−31.8 mm
Ki	22.07	Ki	1.1	y	40 mm	y	[10,30]	y	−40.5 mm
Kd	572.99	Kd	61	z	50 mm			z	−4.70 mm

2.3. Encoder design

In order to track movement, each of the printer axis stepper motors was equipped with custom magnetic rotation encoders. The mounting of the rotation encoder for print extrusion on the print head, as shown in Fig. 1d, required the removal of the cooling fan. The placement of the z-axis encoders required moving the vertical lead screws upward along with the print bed. The print bed was converted to be directly mounted to the y-axis slider, to suit the upward movement of the lead screws, removing the original bed tension springs and their play. The encoder boards were powered through an external lab power supply adjusted to 5 V, making use of 3-pin RCA cable to distribute power and shield the encoder signal. Fig. 4 shows the designed circuit board to fit the AS5600 encoders with the option for I2C and analog readout. The boards are bolted to the back of a servo motor with an appropriate set of spacers, for which a magnet was glued to its shaft, allowing for observation of its relative position.

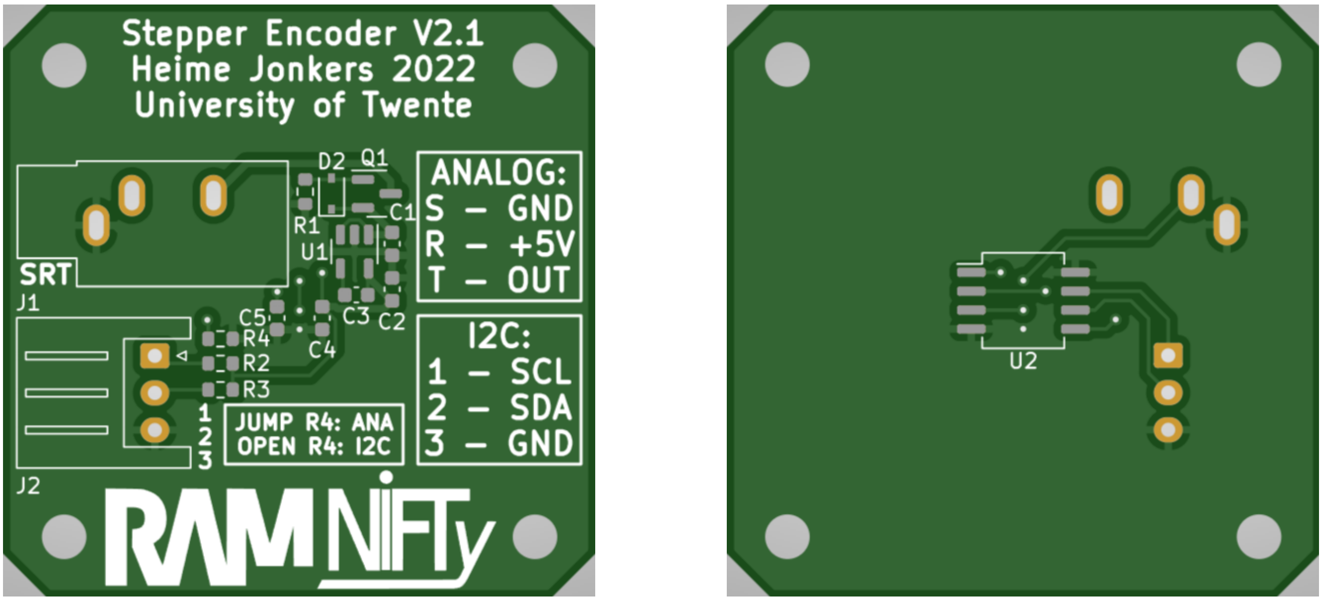

(a) Top side PCB design

(b) Bottom side PCB design

Figure 4. Hall effect rotation encoder shield for stepper motors

In addition to the circuit board layout, Fig. 5 provides the schematic diagram for the custom rotation encoders. The MAX8510 IC provides a regulated 4.5V output voltage for the rotation encoders. The transistor allows for input protection, with the AS5600 providing the rotation encoding. Both an audio jack for the analog output as well as a 2.54 mm header for the I2C output were included to allow for future setup changes. A bill of materials for stepper motor rotation encoders can be found in Table 3

Table 3. Bill of material for stepper motor encoder

Id	Designator	Package	Quantity	Designation
1	C4	C_0603_1608Metric	1	100n
2	D2	D_SOD-323	1	8V2
3	R3,R2	R_0603_1608Metric	2	4k7
4	R1	R_0603_1608Metric	1	1k
5	J2	JST_S3B-XH-A_P2.50mm	1	Conn_01x03_Male
6	Q1	SOT-23	1	SQ2303ES-T1_BE3
7	C3	C_0603_1608Metric	1	10n
8	J1	CUI_SJ1-3533NG_3.5mm	1	AudioJack3
9	C5,C2,C1	C_0603_1608Metric	3	1u
10	R4	R_0603_1608Metric	1	0R
11	U1	SOT-23-5	1	MAX8510EXK45+T
12	U2	SO-8_5.3x6.2mm_P1.27mm	1	AS5600-ASOM

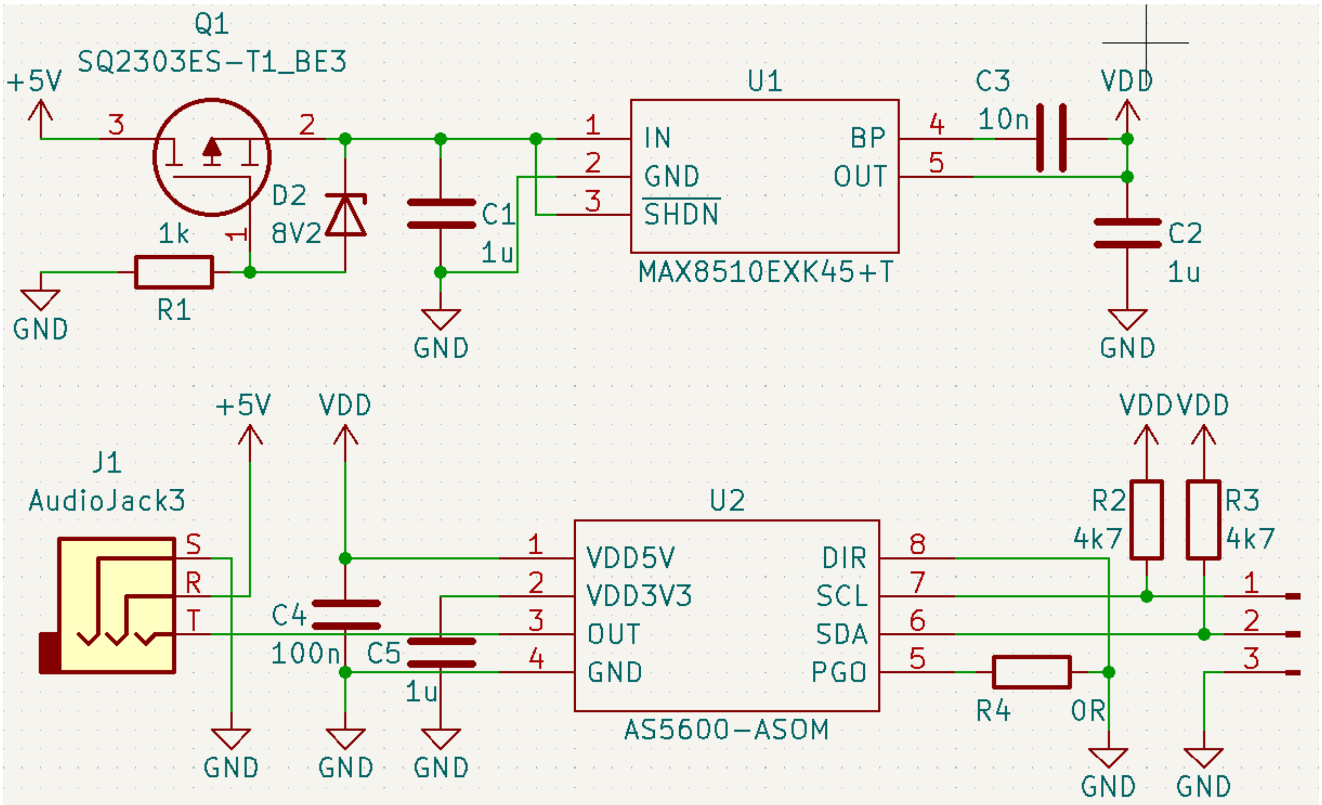

Figure 5. Circuit diagram of the Hall effect rotation encoder shield

3. Samples and Print settings

3.1. Square Tube

The square tube test print, consists of a square, single walled $20\,\mathrm{mm} \times 20\,\mathrm{mm} \times 20\,\mathrm{mm}$ test print designed covering all corner electrodes. It was designed with a wall thickness of $0.4\,\mathrm{mm}$ to match the commonly used $0.4\,\mathrm{mm}$ extrusion nozzle bore. This tube was then printed either as a solid walled tube, a pierced tube with equally spaced 9×9 grid of $0.4\,\mathrm{mm} \times 0.4\,\mathrm{mm}$ holes or a tube pierced by a 4×4 grid of perforations by $2.5\,\mathrm{mm} \times 2.5\,\mathrm{mm}$ squares. The three versions of the tube can be observed in Fig. 6

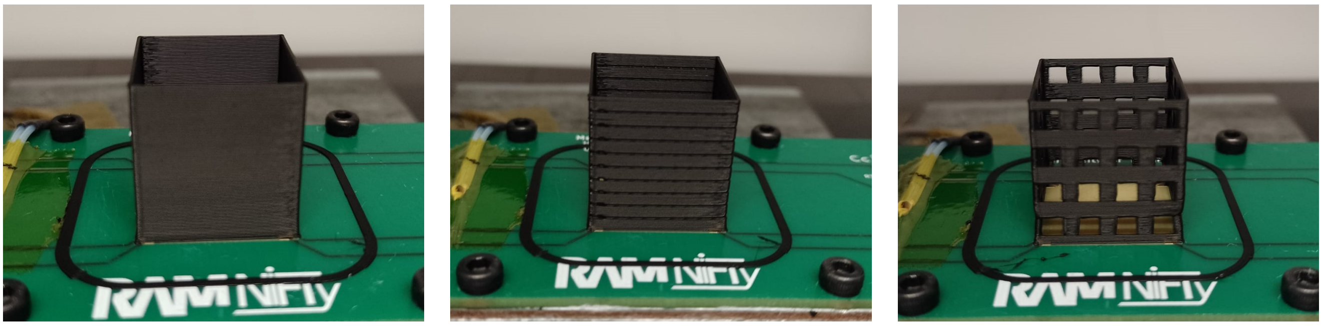

(a) Solid square tube **(b)** Perforated square tube **(c)** Interrupted square tube

Figure 6. Square tube test prints

The square tubes were printed at the same print settings in Protopasta conductive PLA.[2] The settings were based upon manufacturers recommendations.Table 4 provides the setting used in combination Cura slicer 5.2.1.[3]

Table 4. Square tube slicer settings

	Bed	Nozzle	Flow	Speed	Layer height	Nozzle	Cooling	Bed adhesion	Infill
cPLA[2]	60 °C	205 °C	100 %	25 mm s^{-1}	0.2 mm	0.4 mm	No fan	3 line skirt	-

3.2. Benchy

The Benchy 3D printing test[4] standard offers many features relevant to more common 3D print geometries. Amongst these features are overhangs, thin pillars, flat and arched overhangs, flat sections, cylindrical structures and text on the first layer for consistency checking. Given the original Benchy has a maximum dimension of 60 mm, the test print was scaled down to have a length of 20 mm to fit the 20 mm × 20 mm print area.

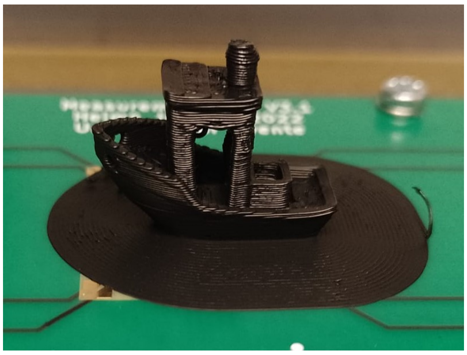

Figure 7. Benchy test print with brim as printed

The scaled print is shown in Fig. 7, including the printed brim. The print settings for the Benchy followed from the manufacturers recommendations of Protopasta conductive PLA,[2] and are provided in table 5. The slicer used was Cura slicer 5.2.1.[3]

Table 5. Benchy slicer settings

	Bed	Nozzle	Flow	Speed	Layer height	Nozzle	Cooling	Bed adhesion	Infill
cPLA[2]	60 °C	205 °C	100 %	40 mm s^{-1}	0.2 mm	0.4 mm	No fan	8 mm brim	Cubic 20%

3.3. Test Object

The test object is an open source calibration design by Raphael Schaaf.[5]

The object is shown in Fig. 8 and consists of several distinct features such as embedded letters on the x, y, z-axis, a cylindrical hole and a matching cylinder and circular and square holes with an overhanging section. The test object was also printed in Protopasta conductive PLA[2] and sliced in Cura slicer 5.2.1[3] in accordance with manufacturer's recommendations as shown in Table 6.

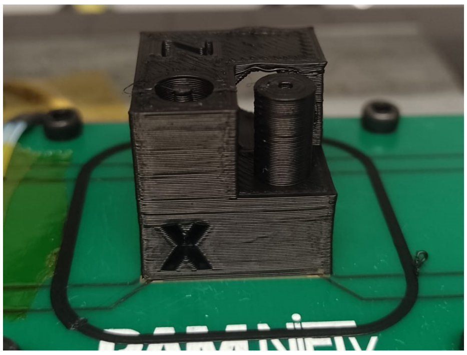

Figure 8. Calibration test object with several geometrical features

Table 6. Test object slicer settings

	Bed	Nozzle	Flow	Speed	Layer height	Nozzle	Cooling	Bed adhesion	Infill
cPLA[2]	50 °C	200 °C	100 %	20 mm s^{-1}	0.1 mm	0.4 mm	No fan	3 line skirt	Cubic 20%

4. Benchy Additional Data

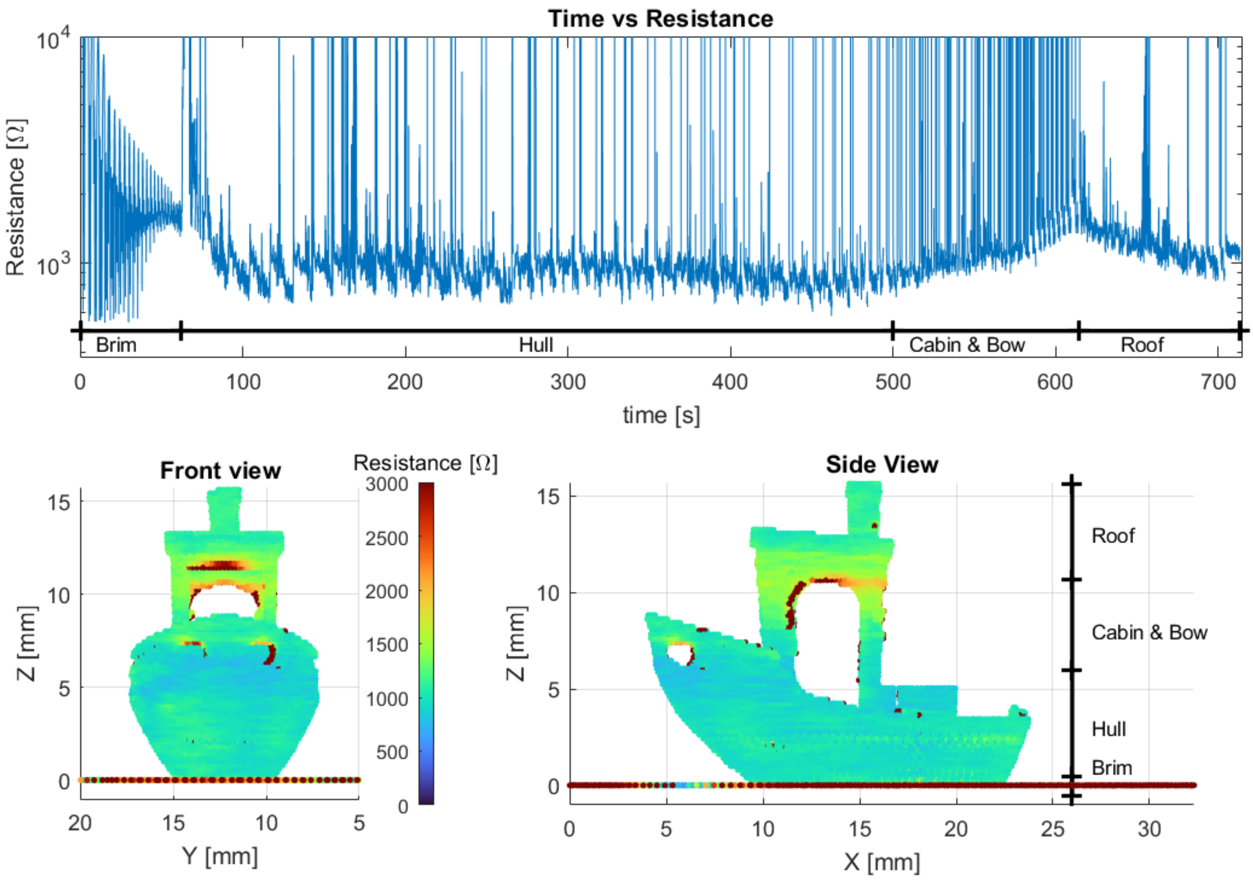

Figure 9. Time and spatial data of multi-electrode measurement of a Benchy print, indicating the different phases of the print. In the resistance plot every phase has a distinctive overall slope and maxima and minima.

This supplementary section presents additional plots from the Benchy multi-electrode measurement data. The resistance time series in Fig. 9 clearly shows the various phases for printing the Benchy geometry, namely the brim, hull, cabin with bow and roof. The large resistance spikes are mainly moments where the nozzle reconnects or disconnects because of travel motion combined with some

defects (where the travel itself is filtered out). It can be noted that printing of the hull first lowers the resistance, whereas printing of the thin cabin pillars increases the resistance and printing of the roof finally lowers the resistance again a bit, because the roof connects the pillars in a parallel fashion. A plotting tool is also developed that can be used to study the individual layers, as shown in Fig. 10. In these plots it becomes clear that the hull perimeter connects well, whereas some of the infill track elements or traxels only connect to the rest of the print at few locations. Locations where bridging occurs can be recognised from the high resistance through the single traxel, for example during bridging of the roof in layer 62.

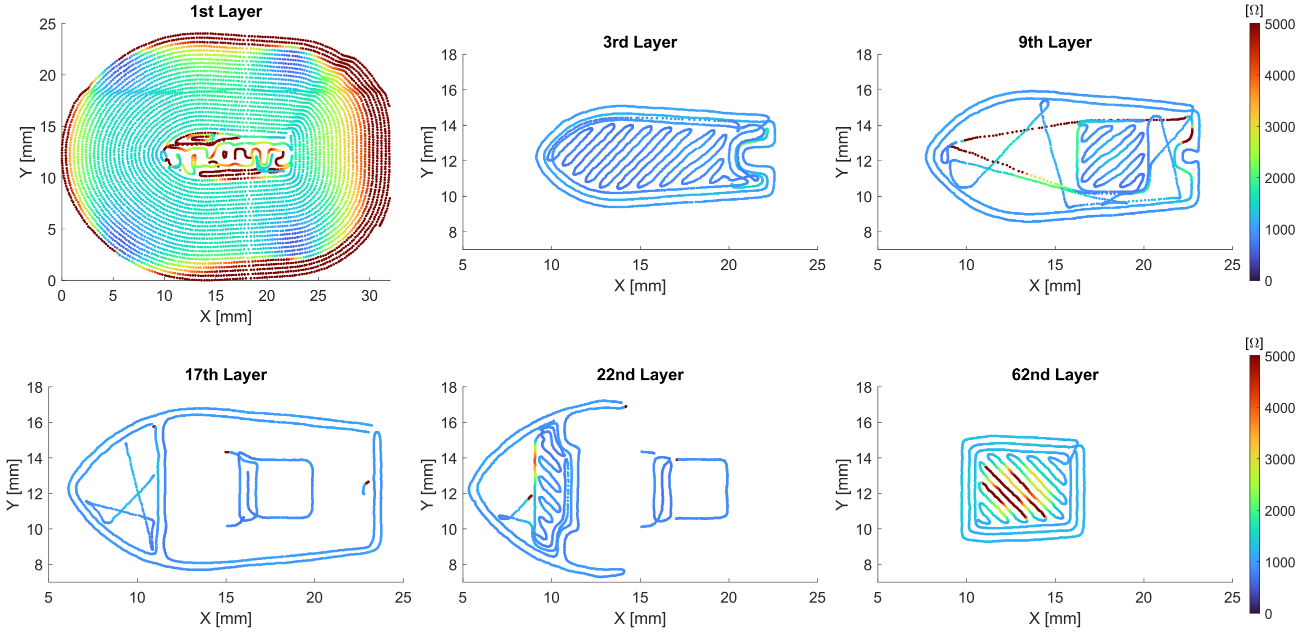

Figure 10. Several layers of the Benchy print, showing the brim in layer 1 and the hull with infill in the layers after. Layer 62 specifically shows the bridging of the roof with its high resistance.

5. Model Explanation

The anisotropic electrical conduction model from the authors[6] is adapted to represent the printing of the square tube geometry. In the following section the main steps are described, to indicate some changes that were implemented with respect to the original model. The model consists of a set of traxels represented as coupled transmission lines (as shown in Fig. 5.a in the main paper), limited to a DC analysis in this research. An example for the square tube geometry is shown in Fig. 11, where the nozzle is printing the third layer. The layers are split in two parts to represent the printing process, section a with the N layers where the nozzle just passed by and section b with the $N-1$ layers for which the nozzle still needs to come along. The total perimeter is $L_{\text{tot}} = L_a + L_b$ long and by sweeping over the ratio of L_a/L_{tot} and the number of layers the model simulates the changing geometry due to the printing process. To implement printing of the third layer ($N = 3$), the model therefore uses $n = 5$ separate transmission lines or traxels in this case ($n := 2N - 1$). These are given the appropriate boundary conditions at the ends to properly link them together and to provide an input and ground.

The voltage in every n^{th} traxel is represented by a single ODE, which can be derived from a network representation with bulk and contact resistances:[6]

$$\frac{\partial^2 U_n(x)}{\partial x^2} + \Gamma_{\text{DC}} \frac{\{-U_{n-1}(x) + 2U_n(x) - U_{n+1}(x)\}}{H^2} = 0 \tag{1}$$

where H is the height of the traxels.

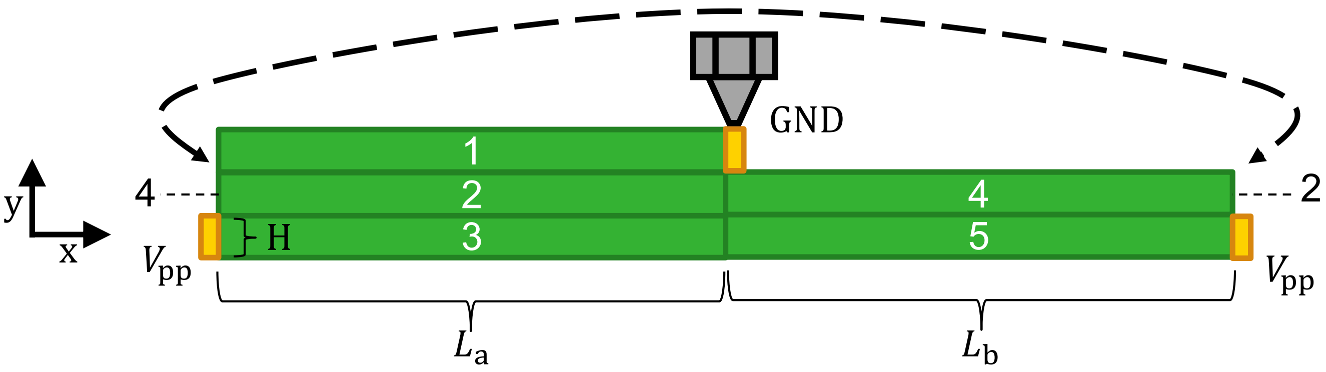

Figure 11. The model lay-out of a square tube, with a corner electrode on the bottle and the moving nozzle on the top. The perimeter is split in two sections, a and b and connected through the boundary conditions. Every traxel has height H.

The anisotropy ratio Γ expresses the ratio in homogenized intra-layer or bulk and inter-layer or contact resistivity, ranging from 0 (only bulk conduction) to 1 (isotropic conduction) for physical parameters:

$$\Gamma_{\text{DC}} = \frac{\rho}{\rho + \sigma/H} \tag{2}$$

With eq. 1 for every traxel a system of equations can be constructed, containing the interaction with the two neighbouring traxels (where the traxels at the bottom and top only have one neighbouring traxel). This system describes a discretized Laplace equation where each printed layer has a lumped voltage in the y-direction. This system of equations can be solved as an eigenvalue problem. The following general voltage solution is expected for the nth traxel:

$$U_n(x) = \sum_{i=1}^{6} C_i e^{\lambda_i x} \tag{3}$$

It should be noted that the traxels in section a can have a different length x compared to section b, due to the nozzle position. For $n = 5$, describing the situation in which the 3rd layer is printed, this results in the following system of equations:

$$\begin{bmatrix} (\lambda^2 + \frac{\Gamma}{W^2}) & -\frac{\Gamma}{W^2} & 0 & 0 & 0 \\ -\frac{\Gamma}{W^2} & (\lambda^2 + 2\frac{\Gamma}{W^2}) & -\frac{\Gamma}{W^2} & 0 & 0 \\ 0 & -\frac{\Gamma}{W^2} & (\lambda^2 + \frac{\Gamma}{W^2}) & 0 & 0 \\ 0 & 0 & 0 & (\lambda^2 + \frac{\Gamma}{W^2}) & -\frac{\Gamma}{W^2} \\ 0 & 0 & 0 & -\frac{\Gamma}{W^2} & (\lambda^2 + \frac{\Gamma}{W^2}) \end{bmatrix} \begin{bmatrix} U_1 \\ U_2 \\ U_3 \\ U_4 \\ U_5 \end{bmatrix} = \begin{bmatrix} 0 \\ 0 \\ 0 \\ 0 \\ 0 \end{bmatrix} \tag{4}$$

It becomes clear that the first three traxels are not coupled to the last two traxels in the system of equations, since they are only connected at the end boundaries. Solving this system results in 10 eigenvalues λ_i and eigenvectors $\vec{\nu}_i$:

$$\vec{U}(x) = \sum_{i=1}^{10} \alpha_i \vec{\nu}_i e^{\lambda_i x} \tag{5}$$

Boundary conditions are required to solve for the constants α_i. The following boundary conditions can be applied:

- Dirichlet (applied voltage): $U_n(x = 0 \vee L) = U_{\text{in/out}}$
- Neumann (applied current): $\frac{\partial U_n(x=0 \vee L)}{\partial x} = -\frac{HW}{\rho} I_{\text{in/out}}$

- Robin (applied resistance): $U_n(x = 0 \vee L) - RI_n(x = 0 \vee L) = U_{\text{in/out}}$
- Periodic (connecting ends of traxel m and n): $U_n(x = 0 \vee L) = U_m(x = 0 \vee L)$ and $\frac{\partial U_n(x=0\vee L)}{\partial x} = -\frac{\partial U_m(x=0\vee L)}{\partial x}$

In the square tube implementation the Dirichlet condition is used for connecting the source and ground and the periodic condition is used for connecting traxel blocks a and b on both ends. For five traxels with each two boundaries, this results in ten equations. This system of equations can be solved to express α_i in terms of the known parameters $\vec{\nu}_i$, λ_i and x.

Since the model does not work for single traxels without neighbouring elements, the Robin condition is used in case of $N = 2$ to connect a linear resistance ($R = \rho L/A$) to section a, where the linear resistance represents section b. In case of $N = 1$ the resistance is simply modelled as linear resistance with nozzle position.

For simplicity the input side in Fig. 11 is defined at the outer edges and the output side is defined at the center. Therefore the positive direction in traxels in section a is from left to right and in section b from right to left, giving rise to the minus sign for the current in the periodic boundary condition. As a consequence the current flowing into the traxels at the outer edges is always defined positive. By rewriting eq. 5 for the two boundary conditions of every traxel, a system of $2n$ equations can be constructed to solve for α_i. This gives the final voltage solution in every location, an example of solving for the boundary conditions can be found in.[6]

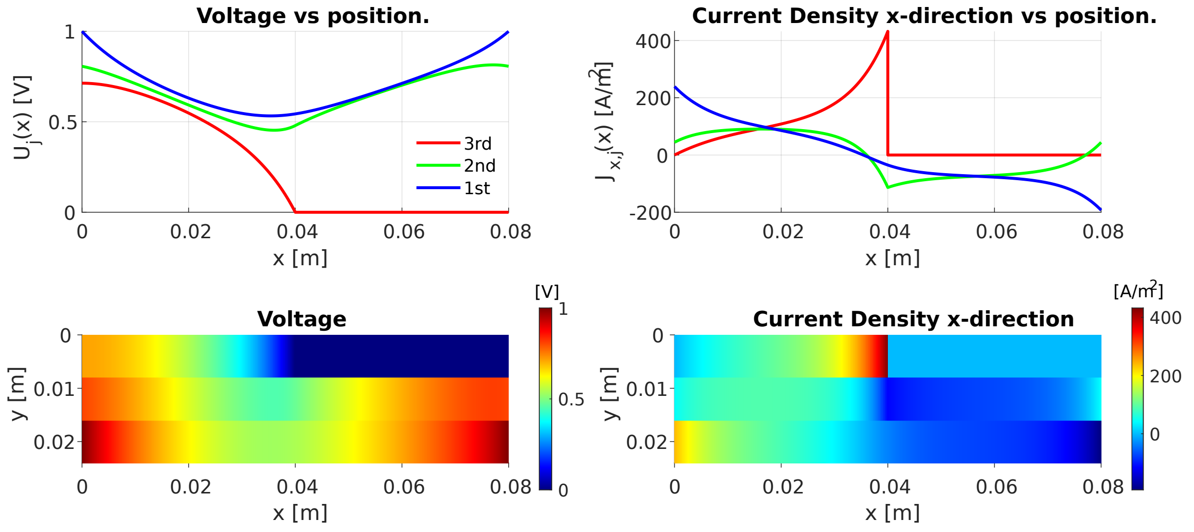

Figure 12. Voltage and x-direction current density plots of the square tube model. The nozzle is halfway printing the third layer, with a single corner electrode at the bottom layer. Exaggerated dimensions and electrical parameters are used to more clearly visualize this example (with $\Gamma = 0.84$).

The current in x-direction is required at the input for calculating the total resistance. It follows from the calculated voltages:[6]

$$I_n(x) = -\frac{HW}{\rho}\frac{\partial U_n(x)}{\partial x} \tag{6}$$

The total resistance can be determined between the input(s) and output(s), where at the input and output the current can only flow in x-direction due to the geometry:

$$R_{\text{total}} = \frac{U_{\text{in}} - U_{\text{out}}}{I_{in}} \tag{7}$$

The corresponding conductance is simply the inverse of the resistance:

$$G = 1/R_{\text{total}} \tag{8}$$

The resulting voltage and current density in x-direction are displayed for every traxel in Fig. 12. The nozzle is positioned on the opposite corner from the electrode, halfway the third layer. From the plot it can be observed that the current spreads out over the entire sample. The model is most accurate in case of large numbers of layers and for more pronounced anisotropy, when lumped circuit models are not accurate.[7] The original code, which was extended to model a square tube, can be found in.[7] The data and adapted code are accessible in.[8]

6. Finite Element Simulations

This section demonstrates the use of the Finite Element Method (FEM) to simulate the in-situ resistance monitoring for a square tube with large perforations and a single corner electrode, Fig. 13. This geometry has a discontinuous print path due to the holes and the use of FEM for the analysis is therefore preferred over the analytical model. The structure is simulated in 2D by FEM using the Electric Currents module of COMSOL. The electrical properties are implemented in COMSOL through the material properties and contact impedance functionality. Like for the analytical model, the tube is simulated in 2D with periodic boundary conditions. The large perforations are simulated as air with a very high electrical resistivity. A parameter sweep is performed over the number of layers and the nozzle position to quasi-statically simulate the printing process. For the material the same parameters are used as for the analytical paper ($\rho = 0.13\,\Omega\,\text{m}$, $\sigma = 3 \times 10^{-6}\,\Omega\,\text{m}^2$, $H = 0.2\,\text{mm}$, $W = 0.4\,\text{mm}$ and $L = 80\,\text{mm}$), with perforations of $2.5\,\text{mm} \times 2.5\,\text{mm}$. The FEM simulations have already been experimentally validated with a mesh convergence study and experiments for the DC-case in previous work.[9]

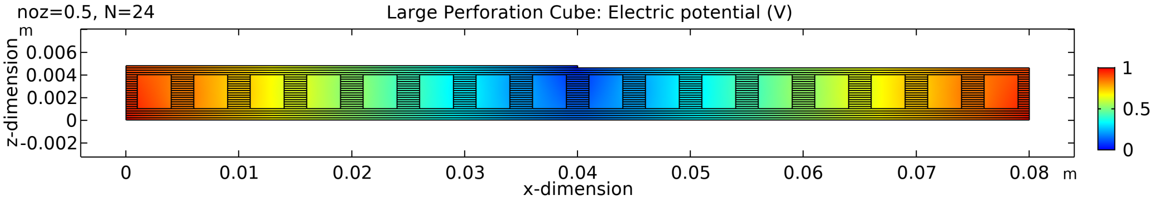

Figure 13. FEM voltage simulation of a perforated tube. The nozzle is at the center of the top layer and the electrodes are on both bottom corners. The large squares are perforations, with the simulation sweeping the top four layers.

The comparison between the FEM result and the measured data for the first four layers after the layers with large perforations is shown in Fig. 14. There is a clear effect of the sixteen holes around the perimeter on the resistance. When the nozzle crosses the perforations for the first time, the resistance monotonically increases after which it suddenly drops when the nozzle crossed a gap. The effect of the gaps is almost negligible for the second layer. From the simulations it actually becomes clear in which direction the nozzle moves due to the asymmetric resistance peaks. The peaks in the simulation are a bit sharper than in reality, where thermal effects and smearing of the material also influence the resistance. Nevertheless the match between FEM and the data shows the feasibility of using FEM to simulate in-situ monitoring of more complex geometries.

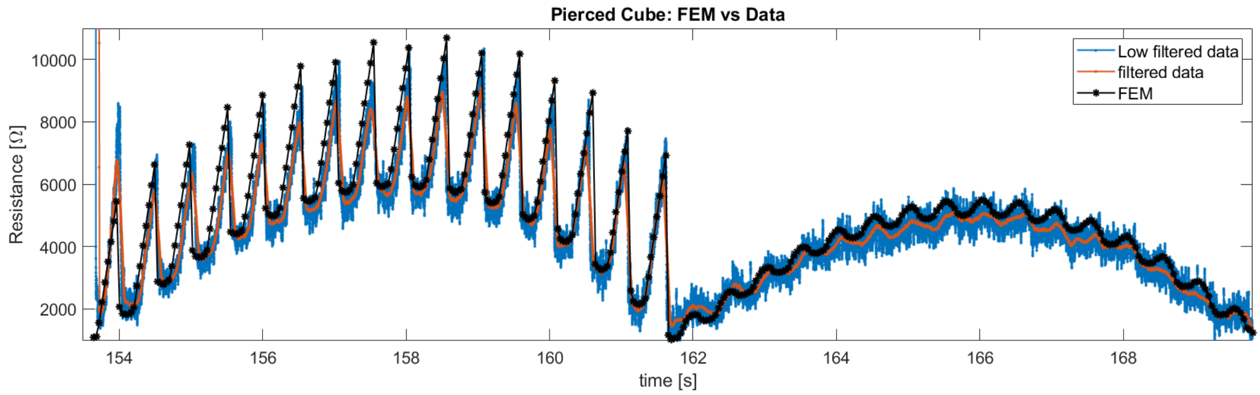

Figure 14. The resistance both from measurements and FEM simulations for the square tube with large perforations for the first two layers on top of the perforations. The measurement data is shown filtered lightly and filtered more to indicate the main features.

7. Required Filament Resistivity

The in-situ monitoring technique is highly scalable due to the large range of measurable resistances which, combined with the scalability of the number of electrodes, allows for an optimum setup for every print bed size.

The resistances are measured with a TiePie HS5 digital oscilloscope, which is able to do resistance measurements in ranges from $0\,\Omega$ to $100\,\Omega$ to a range of $0\,\mathrm{M\Omega}$ to $2\,\mathrm{M\Omega}$ with an accuracy of $1\,\%$ of the full scale. This results in a minimum measurement limit of $1\,\Omega$ and a maximum measurement limit of $2\,\mathrm{M\Omega}$ for the current setup. The observed print resistance is determined by both the material properties (bulk resistivity and inter-layer resistivity), the print settings and the print geometry.

As an indication for the limiting use cases, a single-electrode measurement with a simple geometry can be defined for the upper and lower bound resistance scenarios. The lowest resistance occurs when the nozzle is directly above an electrode while printing the first layer, fig. 15 (left). The highest resistance is achieved when printing a single traxel over the entire width of the bed, taking into account the $220\,\mathrm{mm}$ wide print bed from the Ender S1 Pro, fig. 15 (right). The resistance of a single track of printed material is $R = \frac{\rho L}{HW}$, providing the relation between print geometry and bulk resistivity for a single track of $100\,\%$ infill. Taking a track width of $W = 0.4\,\mathrm{mm}$ and a height of $H = 0.2\,\mathrm{mm}$, we find a lower resistivity limit by reshuffling the resistance expression (where the conduction path is from top to bottom, switching L and H around):

$$\rho_{\text{min}} = \frac{R_{\text{min}} LW}{H} = \frac{1\,\Omega \cdot 0.4 \times 10^{-3}\,\mathrm{m} \cdot 0.4 \times 10^{-3}\,\mathrm{m}}{0.2 \times 10^{-3}\,\mathrm{m}} = 8 \times 10^{-4}\,\Omega\,\mathrm{m} \tag{9}$$

The maximum resistivity can then be derived (where the conduction path runs from left to right, through length L):

$$\rho_{\text{max}} = \frac{R_{\text{max}} HW}{L} = \frac{2 \times 10^{6}\,\Omega \cdot 0.2 \times 10^{-3}\,\mathrm{m} \cdot 0.4 \times 10^{-3}\,\mathrm{m}}{220 \times 10^{-3}\,\mathrm{m}} = 0.73\,\Omega\,\mathrm{m} \tag{10}$$

This gives an estimated required electrical resistivity range of $8 \times 10^{-4}\,\Omega\,\mathrm{m}$ to $0.73\,\Omega\,\mathrm{m}$, almost three orders of magnitude, which can be increased further with a different resistance measurement device (a measurement range up to $20\,\mathrm{M\Omega}$ is not uncommon for commercially available measurement devices). This would increase the achievable range up to $7.3\,\Omega\,\mathrm{m}$. For materials with an even higher resistivity the range could also be increased by adding additional electrodes on the bed, shortening the effective current paths. However, for measurements at the low end of the resistivity, limiting factors such as noise and contact resistance will become significant. The determined resistivity range enables the use of many

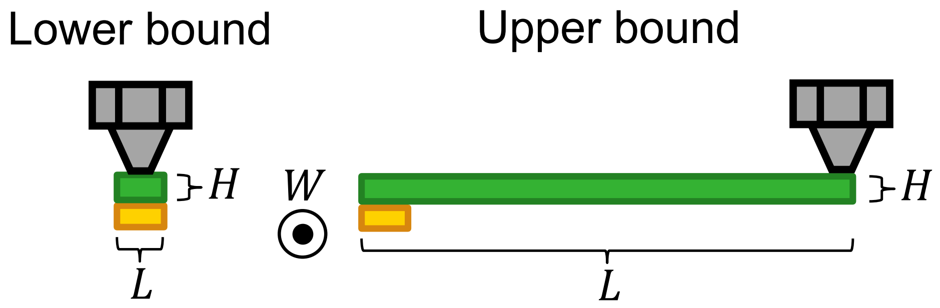

Figure 15. The print geometries for the minimum required resistivity estimate (left) and for the maximum allowed resistivity estimate (right).

commercially available conductive filaments with carbon-based fillers, as shown in table 7. The reported resistivity values are after printing and can be higher during printing due to the positive temperature coefficient of these materials. All carbon-based filaments fall within the hypothetical measurement range, up to $7.3\,\Omega\,\mathrm{m}$. Filaments with metal particles, like the copper nanowires-based Electrifi, have a resistivity that requires more sensitive measurement equipment. Additionally it should be noted that, besides the commercially available filaments, a lot of custom filaments with different fillers have been fabricated in research, which fit within the required resistivity range (e.g.[10,11]).

Table 7. The electrical resistivity for commercially available conductive filaments.

Filament Type	Bulk Resistivity	Conductive-filler	Suitable
ProtoPasta cPLA[2]	$0.15\,\Omega\,\mathrm{m}$	carbon black	yes
AlfaOhm[12]	$0.15\,\Omega\,\mathrm{m}$	carbon nanotubes	yes
Ninjatek EEL cTPU[13]	$0.22\,\Omega\,\mathrm{m}$[14]	carbon black	yes
BlackMagic3D Graphene PLA[15]	$6 \times 10^{-3}\,\Omega\,\mathrm{m}$	graphene	yes
PI-ETPU 85-700+[16]	$2.8\,\Omega\,\mathrm{m}$[9]	carbon black	no
Electrifi[17]	$6 \times 10^{-5}\,\Omega\,\mathrm{m}$	copper	no

References

[1] M. Risco-Castillo, "Professional firmware for the creality ender 3 v2/s1 printers." [Online]. Available: https://github.com/mriscoc/Ender3V2S1

[2] ProtoPasta, *Conductive PLA TDS*, Protoplant Inc., 12001 NE 60th Way B2, Vancouver, Washington, 98682, United States, 2021. [Online]. Available: www.proto-pasta.com/pages/technical-data-sheets

[3] Ultimaker, *Ultimaker Cura*, Ultimaker B.V., Stationsplein 32, 3511 ED, Utrecht, The Netherlands, 2021. [Online]. Available: www.ultimaker.com/software/ultimaker-cura

[4] "3D Benchy," 2020, https://www.Creative-Tools.com is licensed under a Creative Commons Attribution-NoDerivatives 4.0 International License. Permissions beyond the scope and typical usage examples are explained at https://www.3dbenchy.com/license/. [Online]. Available: https://https://www.3dbenchy.com/

[5] "Filament Test Cube," 2017, https://www.thingiverse.com/thing:2166102 This work is licensed under the Creative Commons - Attribution - Share Alike license. To view a copy of this license, visit https://creativecommons.org/licenses/by-sa/4.0/deed.en. [Online]. Available: https://www.thingiverse.com/thing:2166102

[6] A. Dijkshoorn, M. Schouten, S. Stramigioli, and G. Krijnen, "Modelling of anisotropic electrical conduction in layered structures 3d-printed with fused deposition modelling," *Sensors*, vol. 21, no. 11, 2021.

[7] ——, "Model code of anisotropic electrical conduction in layered 3d-prints with fused deposition modeling," 2021, http://doi.org/10.4121/14364710.V1.

[8] H. Jonkers, A. Dijkshoorn, S. Stramigioli, and G. Krijnen, "Data and code of fff print defect characterization through in-situ electrical resistance monitoring," 2024, http://doi.org/10.4121/c17ac579-bab1-4f58-ba67-a95182fd021f.

[9] A. Dijkshoorn, M. Schouten, G. Wolterink, R. Sanders, S. Stramigioli, and G. Krijnen, "Characterizing the electrical properties of anisotropic, 3d-printed conductive sheets for sensor applications," *IEEE Sensors Journal*, vol. 20, no. 23, pp. 14 218–14 227, 2020.

[10] S. W. Kwok, K. H. H. Goh, Z. D. Tan, S. T. M. Tan, W. W. Tjiu, J. Y. Soh, Z. J. G. Ng, Y. Z. Chan, H. K. Hui, and K. E. J. Goh, "Electrically conductive filament for 3d-printed circuits and sensors," *Applied Materials Today*, vol. 9, pp. 167–175, 2017. [Online]. Available: https://www.sciencedirect.com/science/article/pii/S235294071730152X

[11] C. J. Hohimer, G. Petrossian, A. Ameli, C. Mo, and P. Pötschke, "3D Printed Conductive Thermoplastic Polyurethane/Carbon Nanotube Composites for Capacitive and Piezoresistive Sensing in Soft Pneumatic Actuators," *Additive Manufacturing*, p. 101281, 2020.

[12] Maip Compounding S.r.l., *ALFAOHM*, FILOALFA, 2024. [Online]. Available: https://www.filoalfa3d.com/gb/content/20-alfaohm

[13] NinjaTek, *NinjaTek Eel 3D Printing Filament TDS*, Fenner Inc., 311 W Stiegel Street, Manheim, Pennsylvania, 17545, USA, 2021. [Online]. Available: www.ninjatek.com/eel

[14] H. Jonkers, "3d printed electronics," MSc thesis, University of Twente, 2022, http://essay.utwente.nl/89406/.

[15] BlackMagic3D, *Conductive graphene PLA filament*, Graphene Laboratories Inc., 760 Koehler Avenue - Suite 2, Ronkonkoma, United States, NY 11779, 2017. [Online]. Available: www.web.archive.org/web/20170610125904/http://www.blackmagic3d.com/Conductive-p/grphn-pla.htm

[16] Palmiga Innovations, *ETPU-95 Carbon Black*, Palmiga Innovations, Fläderbärsvägen 4, 263 71, Jonstorp, Sweden, 2016. [Online]. Available: www.rubber3dprinting.com/pi-etpu-95-250-carbon-black/

[17] Multi3D, "Electrifi Conductive Filament." [Online]. Available: https://www.multi3dllc.com/product/electrifi/
